# Community interventions with women’s groups to improve women’s and children’s health in India: a mixed-methods systematic review of effects, enablers and barriers

**DOI:** 10.1136/bmjgh-2020-003304

**Published:** 2020-12-15

**Authors:** Sapna Desai, Madhavi Misra, Aikantika Das, Roopal Jyoti Singh, Mrignyani Sehgal, Lu Gram, Neha Kumar, Audrey Prost

**Affiliations:** 1 Population Council India, New Delhi, Delhi, India; 2 International Food Policy Research Institute, New Delhi, India; 3 Institute for Global Health, University College London, London, UK; 4 International Food Policy Research Institute, Washington, DC, USA; 5 University College London Institute of Child Health, London, UK

**Keywords:** public health, systematic review, prevention strategies

## Abstract

**Introduction:**

India is home to over 6 million women’s groups, including self-help groups. There has been no evidence synthesis on whether and how such groups improve women’s and children’s health.

**Methods:**

We did a mixed-methods systematic review of quantitative and qualitative studies on women’s groups in India to examine effects on women and children’s health and to identify enablers and barriers to achieving outcomes. We searched 10 databases and included studies published in English from 2000 to 2019 measuring health knowledge, behaviours or outcomes. Our study population included adult women and children under 5 years. We appraised studies using standard risk of bias assessments. We compared intervention effects by level of community participation, scope of capability strengthening (individual, group or community), type of women’s group and social and behaviour change techniques employed. We synthesised quantitative and qualitative studies to identify barriers and enablers related to context, intervention design and implementation, and outcome characteristics.

**Findings:**

We screened 21 380 studies and included 99: 19 randomised controlled trial reports, 25 quasi-experimental study reports and 55 non-experimental studies (27 quantitative and 28 qualitative). Experimental studies provided moderate-quality evidence that health interventions with women’s groups can improve perinatal practices, neonatal survival, immunisation rates and women’s and children’s dietary diversity, and help control vector-borne diseases. Evidence of positive effects was strongest for community mobilisation interventions that built communities’ capabilities and went beyond sharing information. Key enablers were inclusion of vulnerable community members, outcomes that could be reasonably expected to change through community interventions and intensity proportionate to ambition. Barriers included limited time or focus on health, outcomes not relevant to group members and health system constraints.

**Conclusion:**

Interventions with women’s groups can improve women’s and children’s health in India. The most effective interventions go beyond using groups to disseminate health information and seek to build communities’ capabilities.

**Trial registration number:**

The review was registered with PROSPERO: CRD42019130633.

**Key questions**

**What is already known?**

- Women’s groups are widely engaged in health promotion to improve women and children’s health in India and other countries.
- There is little evidence on the effects of different kinds of women’s groups interventions on women’s and children’s health in India, which social and behaviour change strategies work best and for what, and barriers and enablers to effectiveness.

**What are the new findings?**

- Moderate-quality evidence for health interventions with women’s groups indicates positive effects on perinatal practices, neonatal survival, immunisation rates, women’s and children’s dietary diversity and the control of vector-borne diseases in India.
- We found no effects of interventions where groups tackled outcomes influenced by strong social and service-related constraints, such as violence against women, or women and children’s nutritional status.
- Effective women’s groups were open to other community members, inclusive of the most concerned and vulnerable, and had adequate intensity and facilitator capacity.

**What do the new findings imply?**

- Working with women’s groups can improve women’s and children’s health in India if the health outcomes selected are relevant to group members, multiple social and behaviour change techniques are used beyond knowledge transfer and sufficient intervention intensity is achieved.
- Providing health information to existing financial groups may modify some behaviours among group members but does not emerge as an effective approach to improving ‘hard’ health outcomes such as neonatal mortality or women and children’s nutritional status at a group or population level.
- Population-level health improvements through women’s groups require further scale up of community mobilisation interventions that go beyond using groups as a platform to disseminate health information and improve communities’ capabilities.

## Introduction

Community interventions are key to achieving the Sustainable Development Goals for health, nutrition and gender equality.^1 2^ Interventions to improve women’s and children’s health can engage with groups to strengthen the capabilities of individuals, groups and communities to adopt beneficial health practices and shape the social determinants of health.^3–5^ Women’s groups vary in size, membership and goals but typically hold regular meetings for financial savings or livelihoods promotion, health training and action, or a combination. Women’s groups can be ‘closed’, i.e., restricted to members who fulfil specific criteria, for example, those who make financial contributions, or ‘open’ to all women and other community members, in which case they are akin to community groups.^6–9^ Some community interventions use existing groups as a platform to share health information or seek to leverage group cohesion to improve members’ health.^10^ Others aim to improve population health through community mobilisation, defined as ‘a capacity building process through which community members, groups or organizations plan, carry out, and evaluate activities in a participatory and sustained basis to improve their health and other conditions’.^11^

The Government of India currently has two large-scale community engagement initiatives involving women’s groups. The National Rural Livelihoods Mission (NRLM) supports self-help groups (SHGs) engaged in savings, credit and livelihoods promotion. The NRLM has reached over 50 million households by 2020 and aims to reach 70 million by 2025. Capitalising on this coverage, the NRLM introduced health, sanitation and nutrition education into its SHG activities in 2017.^12^ The second government initiative, led by the National Health Mission, incentivises around 1 million community health volunteers called Accredited Social Health Activists (ASHA), to facilitate regular meetings with women’s groups. Meetings are open to all and offer health-related interventions and linkages to public health services.^13^

Despite the extraordinary scale of women’s groups initiatives in India, there has been no review of their effects on women’s and children’s health or factors that can improve implementation.^7–9 14^ We aimed to: (1) review experimental studies that examined the effect of women’s groups interventions with or without a health component on women’s and children’s health in India, compared with either women’s groups without a health intervention or no exposure to a women’s group and (2) identify barriers and enablers related to contextual factors, intervention design and implementation, and outcome characteristics that explain these effects, through a synthesis of qualitative and quantitative studies.

## Methods

### Design, inclusion and exclusion criteria

We conducted a mixed-methods systematic review and included:

- Studies on women’s groups in India, published in English between 1 January 2000 and 31 December 2019.
- Randomised controlled trials (RCTs); non-randomised studies of interventions—referred to here as quasi-experimental studies—with both strong and weaker designs, including studies using difference-in-difference approaches, interrupted time series, regression discontinuity, instrumental variable estimation and propensity score matching;^15 16^ and non-experimental quantitative and qualitative studies.
- Studies of women’s groups that examined health knowledge, behaviours or outcomes, including general illness, Reproductive, Maternal, Newborn and Child Health (RMNCH), nutrition, sexual health and HIV, mental health, communicable and non-communicable disease and violence against women.

We excluded studies that were not conducted in India, reported no empirical data, did not focus on health outcomes or focused on groups where adult women were not primary members. Our study population included all women aged 18 years and above and children under 5 years.

### Literature search and quality appraisal

Two researchers (AP and MM) searched PubMed, SCOPUS, POPLINE, PsycINFO, OpenGrey, Social Sciences Citation Index, International Bibliography of the Social Sciences, 3ie Database of Impact Evaluations, Global Health and Econlit. Online supplemental table 1 lists the search terms. MM and MS screened titles and abstracts, then consulted two expert advisors and four coauthors (AP, LG, NK and SD) to identify other relevant studies. After completing the first round in March 2019, we updated the search to include studies published between April and December 2019. Six researchers (AD, AP, MM, MS, RJS and SD) extracted data on study characteristics, interventions, effects, enablers and barriers and conducted quality appraisals using the Revised Cochrane Risk of Bias for randomised trials, the Risk of Bias in Non-randomised Studies of Interventions and an adapted version of the Critical Appraisal Skills Programme (CASP) for qualitative studies.^17–19^ The review refers to studies as high-quality, moderate-quality or low-quality evidence to reflect the Risk of Bias (RoB) assessment: high quality indicates low RoB; moderate quality indicates some concerns/moderate RoB; and low quality signals high, serious or critical RoB. Two coauthors (AP and SD) independently reviewed all data extracted, compared quality assessments and drafted the synthesis.

10.1136/bmjgh-2020-003304.supp1 Supplementary data

### Synthesis

Our synthesis followed three steps. First, we tabulated the effects of women’s groups across health domains that emerged from experimental studies, irrespective of study quality: (1) RMNCH; (2) nutrition; (3) violence against women; (4) vector-borne diseases; (5) sexual health and HIV; (6) water, sanitation and hygiene; (7) mental health; (8) health expenditure; or (9) multiple domains. Studies were classified by primary outcome domain(s) for RCTs, or main health outcome(s) for quasi-experimental studies. We did not do a meta-analysis or subanalyses as study types and outcomes were highly heterogenous.

Second, we used harvest plots to examine results of high-quality or moderate-quality experimental studies for domains with more than three studies (n=21), along three dimensions as described in box 1: level of community participation, scope of capability strengthening and underlying group type.^20^ Next, we identified social and behaviour change techniques employed in moderate-quality or high-quality studies of interventions with a health component (19/27 studies). We used a taxonomy developed by Kok *et al* to synthesise these in a heat map.^21^ The taxonomy categorises 14 types of techniques that broadly fall into two groups: those aimed at individual knowledge, capacity and skills (eg, using imagery and modelling behaviours) and those aimed at addressing social and environmental conditions (eg, mobilising social networks, participatory learning and action).^21^ We chose this taxonomy because it incorporated more group techniques than others.^22^

Box 1 **Panel 1: dimensions used to examine group interventions to improve health**

Level of community participation*

Drawing on Arnstein,^118^ we identified three main levels of community participation:

- *Informed*: groups have little input into intervention priorities or actions; policymakers or implementing organisations choose the health domain and approach.
- *Consulted*: groups and/or other community members are involved in defining intervention priorities and actions, for example, through formative research.
- *Partnership*: groups and/or other community members define intervention priorities and/or actions.

Scope of capability strengthening*

Drawing on Labonte and Laverack’s work and the 2017 WHO Global Strategy for Women’s, Children’s and Adolescents’ health, we differentiated between four levels of capability strengthening for health:^119^

- *Individual*, for example, individual-level skills training with no emphasis on the group as an enabler.
- *Group*, for example, group-based health education, with little to no attempt to benefit non-group members.
- *Community*, for example, community mobilisation to improve health for group members and non-members.
- *None,* for example, groups that work together on finance, but no specific intention to build individual, group or community capabilities for health.

Underlying group type*

Based on studies in the review, we identified four different types of groups taking part in health interventions:

- *SHGs* primarily engaged in savings and credit activities, with membership restricted to 10–12 women who contribute financially.
- *Community-based women’s groups* for women only, with no other membership requirements.
- *Open women’s groups* that held meetings open to all women and other community members.
- *Special population groups*, for example, female sex worker collectives.

*We separate these three dimensions as they do not necessarily overlap. For example, the underlying group type does not necessarily prescribe the scope of capability strengthening or level of community participation. Similarly, a normally ‘closed’ SHG can open up to non-SHG members to identify and implement community-wide strategies to improve health.

Finally, we developed a summary of enablers and barriers related to contextual factors, intervention design and implementation, and outcome characteristics. Examples of contextual factors were rural/urban geography or migration levels. Implementation factors included types of group facilitator, behaviour change approach and the functioning of the underlying group. Outcome characteristics referred to the specific aim of the intervention, its relevance and feasibility specific to women’s groups, such as whether pregnancy information would be relevant to older members of an SHG.

We described the results of all experimental studies after indicating their risk of bias. For all subsequent syntheses, however, we included only high-quality and moderate-quality experimental studies, along with qualitative studies and quantitative non-experimental studies that met basic criteria in the CASP checklist,^19^ that is, clearly reported methods and data pertinent to our research questions. We employed a results-based convergent synthesis approach^23^: we integrated results from quantitative and qualitative analyses during a final synthesis using a thematic matrix and through iterative review and discussion with coauthors. We present results using the Preferred Reporting Items for Systematic Review and Meta-Analysis Protocols and Synthesis Without Meta-analysis guidelines.^24^ The review is registered with PROSPERO (CRD42019130633). The study was funded by the Bill and Melinda Gates Foundation, who had no role in data analysis, interpretation or writing.

## Results

**Figure 1:**
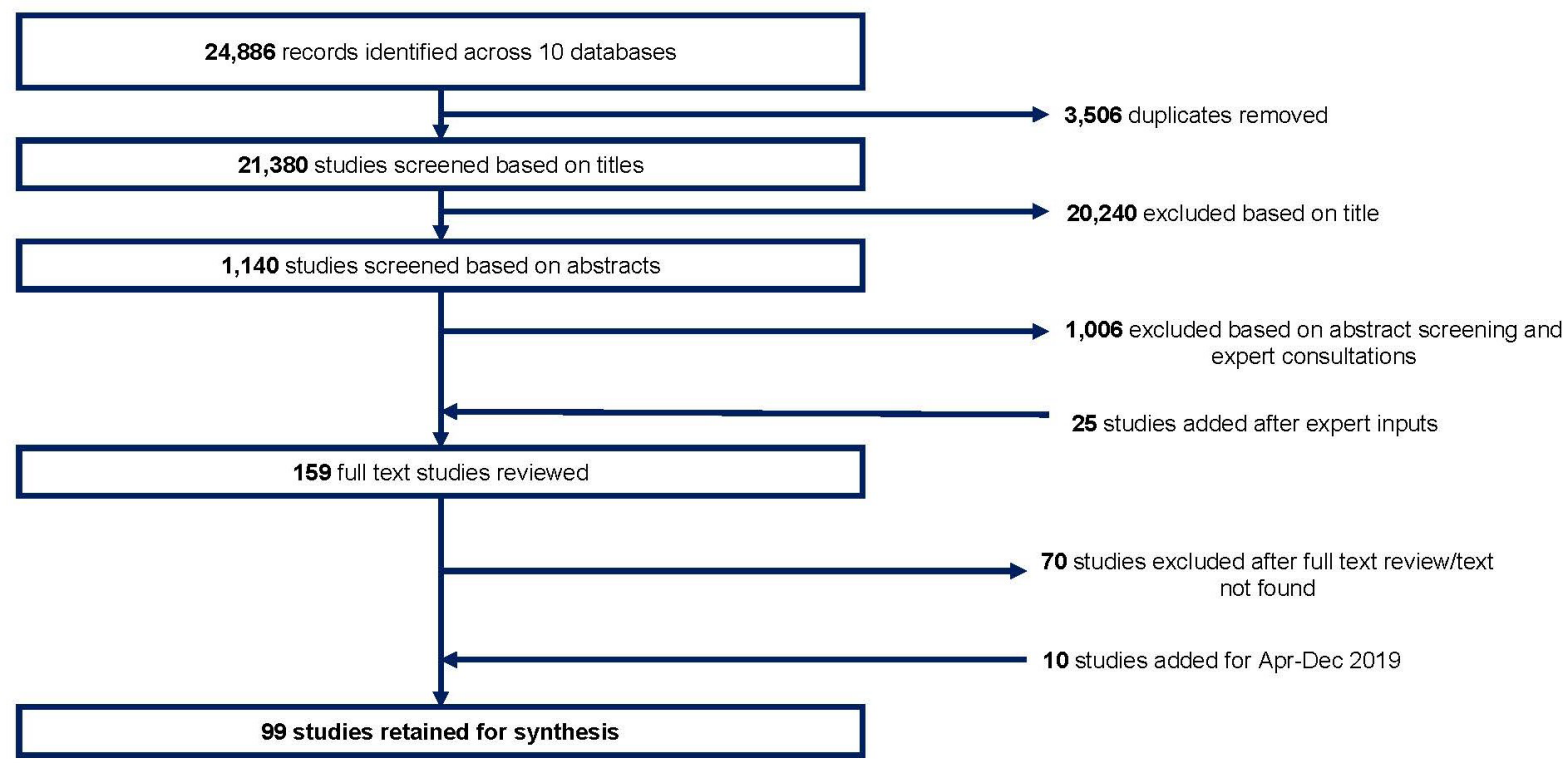
Study selection.

We screened 21 380 studies and included 99 (figure 1). We found 19 RCT reports (17 unique trials and two subanalyses), 25 quasi-experimental study reports (24 unique studies and one subanalysis) and 55 non-experimental studies (27 quantitative and 28 qualitative). Online supplemental figure 1 describes the geographical location of studies, by state. Online supplemental table 2 describes study settings, interventions and their characteristics, type of control, participant inclusion criteria, outcome measure(s), effect size and risk of bias assessment for all RCTs. Online supplemental table 3 describes all quasi-experimental studies. Online supplemental table 4 describes all non-experimental studies.

One-third (17/44) of experimental studies were at high, serious or critical risk of bias (4/19 RCTs and 13/25 quasi-experimental studies). Twenty-seven experimental studies reported on population-level outcomes, 15 reported outcomes only among group members and 2 studies reported outcomes for members and non-members separately. Over 85% of non-experimental studies (24/27 quantitative and 24/28 qualitative) were appraised as relevant and of good quality.

We present results related to our two objectives. First, we describe the effects of women’s groups interventions within health domains and also according to level of community participation, scope of capacity strengthening, type of group and the type of social and behaviour change techniques used. Second, we map enablers and barriers related to context, intervention design and implementation, and outcome characteristics.

### Intervention effects

#### Reproductive, maternal, newborn and child health

Seventeen studies (five unique RCTs, nine unique quasi-experimental studies and three subanalyses) reported on interventions to improve RMNCH. Kumar *et al* did a moderate-quality RCT of a community-wide behaviour change intervention with group meetings and home visits to improve birth outcomes in one rural sub-district.^25^ They found a large reduction in neonatal mortality (adjusted risk ratio: 0.46, 95% CI 0.35 to 0.60) and improvements in maternal care-seeking behaviours.^25 26^ Acharya *et al*^27^ tested a similar behaviour change strategy in a moderate-quality RCT across seven districts, with a less intensive approach. The trial found some improvements in selected perinatal preventive and care-seeking behaviours but no effect on neonatal survival (adjusted OR (aOR): 0.98, 95% CI 0.80 to 1.19).

Two moderate-quality to high-quality RCTs and a moderate-quality quasi-experimental study tested community mobilisation through women’s groups practising participatory learning and action to identify and address problems in the perinatal period with support from the wider community. This approach, including one implemented by ASHAs in five districts, led to reductions in neonatal mortality of around 30% (aOR 0.68, 95% CI 0.59 to 0.78; aOR 0.69, 95% CI 0.53 to 0.89; aOR: 0.69, 95% CI 0.57 to 0.83), with greater reductions among more marginalised families (aOR: 0.41, 95% CI 0.28 to 0.59).^28–31^ A moderate-quality trial of a similar perinatal intervention in Mumbai found no effects on neonatal mortality (aOR 1.42, 95% CI 0.99 to 2.04) or other birth outcomes.^32^ Seven quasi-experimental study reports, of which six were at serious or critical risk of bias, tested adding health information to SHGs in rural settings to improve behaviours in the perinatal period.^33–39^ They reported increases in knowledge of perinatal danger signs, selected essential newborn care and care-seeking practices among group members, but none measured birth outcomes.

Finally, two moderate-quality quasi-experimental studies focused on RMNCH beyond the perinatal period. One tested the impact of community-based women’s groups engaging in collective action based on identified needs in three rural districts, leading to improvements in child immunisation rates (diphtheria pertussis tetanus: coefficient (β): 0.088, SE: 0.037; measles β: 0.076, SE: 0.038; tuberculosis: 0.071, SE: 0.038).^40^ The other evaluated the effects of SHG membership with no health intervention in five districts and found no effects on assisted delivery, breastfeeding and child immunisation rates, knowledge of diarrhoea treatment or family planning.^41^

#### Nutrition

Three RCTs and four quasi-experimental studies focused on nutrition. One high-quality RCT found that giving information about key practices for maternal and child nutrition to SHG members had a small effect on child dietary diversity (mean number of food groups consumed) for the youngest child in the family (β: 0.286, SE: 0.118), but not the index child (β: 0.169, SE: 0.080), and no effects on maternal body mass index (β: −0.025, SE: 0.082).^42^ A high-quality trial of participatory learning and action with groups and home visits to improve child growth reported no improvement in child length-for-age (adjusted mean difference 0.11, 95% CI -0.01 to 0.23) or weight-for-age and weight-for-height z scores, despite increases in maternal and child dietary diversity.^43^ A third RCT found effects of SHGs with no health intervention on child weight-for-height z scores (adjusted β=0.38, 95% CI 0.16 to 0.61) but was at high risk of bias.^44^ Two moderate-quality quasi-experimental studies found that SHG membership with food or livelihood inputs improved energy (109 kcal/day, p≤0.05) and protein intake (5.84 g/day, p≤0.01) for participants in a state-wide programme.^45 46^ A third quasi-experimental study reported lower levels of underweight among the children of SHG members and higher protein intake for their households but was at serious risk of bias.^47^ Finally, a moderate-quality quasi-experimental study testing participatory learning and action with women’s groups combined with home visits and creches with meals for children under 3 years in five blocks found reductions in wasting, underweight and stunting (aOR: 0.73, 95% CI 0.55 to 0.97; aOR 0.60, 95% CI 0.47 to 0.75 and aOR 0.73, 95% CI 0.57 to 0.93, respectively).^48^

#### Violence against women

We identified two RCTs and two quasi-experimental studies on violence against women. Both moderate-quality RCTs evaluated interventions providing gender-transformative training sessions to SHGs.^49 50^ The first, a rural trial, found no improvements in attitudes to gender roles (aOR: 0.69, 95% CI 0.35 to 1.02) or levels of physical marital violence (aOR: 0.69, 95% CI 0.46 to 1.02) and an increase in emotional marital violence (aOR: 2.95, 95% CI 1.75 to 4.97) among members.^49^ The second, an urban RCT, found no effects on experience of physical or sexual violence (β: −0.006, SE: 0.022).^50^ Two quasi-experimental studies examined the effect of SHG membership with no violence-specific intervention: one, a moderate-quality study, found no effect on an index of violence (β: 0.092, SE: 0.074), while the other, a low-quality study, found a small reduction in a similar index (difference-in-difference estimate: −0.448, p=0.008).^51 52^

#### Vector-borne diseases

Two RCTs and one quasi-experimental study tested interventions to prevent vector-borne diseases. A moderate-quality RCT of an urban intervention to educate group members to control dengue found significant reductions in pupae per household and pupae per person indexes (difference in difference in % reduction from baseline: −14.7, p=0.01 and −0.35, p=0.02).^53^ A moderate-quality RCT of a rural community mobilisation intervention engaging group and community members for malaria control reported increases in the proportion of people sleeping under bed nets and receiving prompt diagnosis from a trained provider for a fever (aOR: 1.27, 95% CI 1.14 to 1.42 and aOR 1.45, 95% CI 1.09 to 1.94, respectively).^54^ Finally, one low-quality quasi-experimental study tested the effect of group-led health education and monitoring households to control lymphatic filariasis in two rural villages and found a significant reduction in the proportion of people reporting mosquito-borne diseases (intervention: 75.8%, control: 48.8%, p=0.05).^55^

#### Sexual health and HIV

All but one study that tested group interventions to improve sexual health and reduce sexually transmitted infection (STI)/HIV incidence (n=6) were conducted with female sex workers. One low-quality RCT reported improved HIV knowledge among rural SHG members exposed to a health education intervention (aOR for ‘ever heard of HIV’: 3.6, 95% CI 1.6 to 8.0).^56^ A low-quality RCT among urban sex workers tested introduction of a microenterprise intervention with ongoing health education to reduce the number of sex exchange partners and reported positive results (reduction in partners β: −1.8 (−2.9, 95% CI −2.9 to −0.8).^57^ A moderate-quality quasi-experimental study that examined the effect of community mobilisation interventions with urban and rural sex workers reported reductions in gonorrhoea/chlamydia (aOR: 0.53, 95% CI 0.31 to 0.87), but not on HIV or syphilis (aOR: 1.07, 95% CI 0.54 to 2.14, aOR: 0.63, 95% CI 0.22 to 1.78, respectively), and improvements in condom use and HIV testing.^58^ Another moderate-quality quasi-experimental study evaluated a community mobilisation intervention and reported improved knowledge of STI/HIV (know at least one STI: aOR: 48.5, 95% CI 14.4 to 163) and an overall effect on summary measures of empowerment and health (parameter estimate 4.81 (SE: 0.34), p<0.001).^59^ A moderate-quality evaluation of community mobilisation and peer groups reported reductions in gonorrhoea and/or chlamydia (aOR: 0.60, 95% CI 0.47 to 0.78) but no change in syphilis (aOR: 0.74, 95% CI 0.58 to 0.94) or HIV infection (aOR: 0.89, 95% CI 0.74 to 1.07).^60^ Lastly, a low-quality quasi-experimental study reported positive effects of group training grounded in cognitive behavioural therapy on adherence to antiretroviral therapy (intervention: 54%; control: 0%).^61^

#### Domains with less than three studies

We found less than three studies on: health expenditure,^62 63^ water and sanitation^64 65^ and mental health,^28 66^ as well as two studies that addressed multiple health domains^67 68^ (detailed findings reported in online supplemental text).

#### Effects by level of community participation, scope of capability strengthening and group type

**Figure 2:**
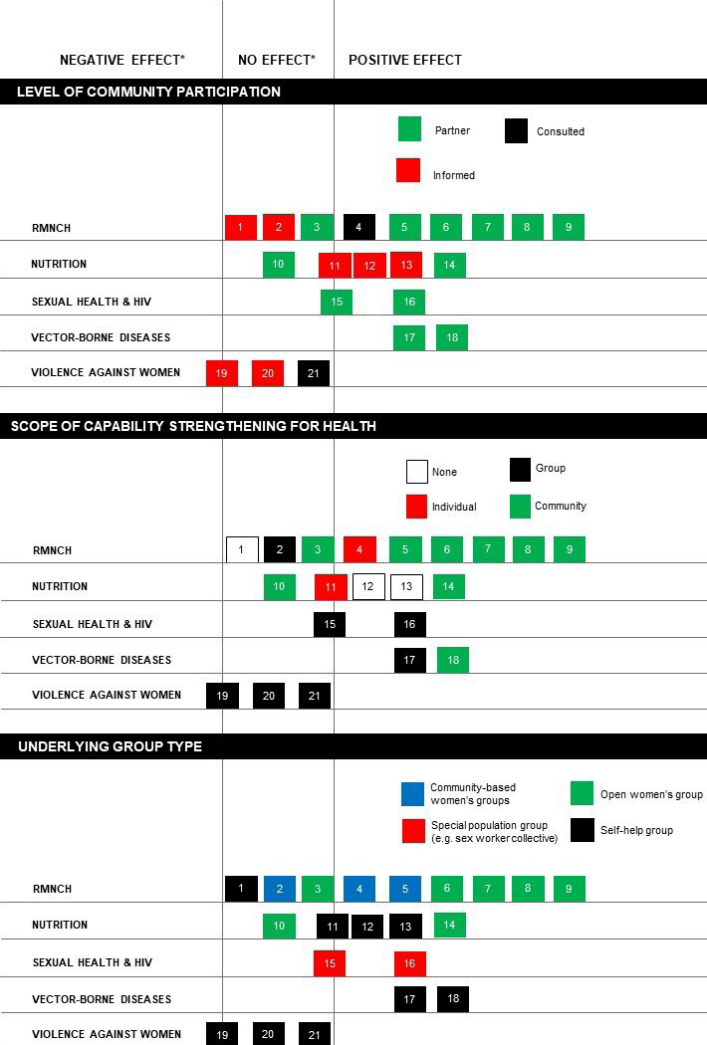
Harvest plots key to studies (first author):1. Prennushi^41^ 2. Acharya^27^ 3. More^32^ 4. Saha^38^ 5. Janssens^40^ 6. Tripathy^28^ 7. Tripathy^29^ 8. Kumar^25^ 9. Roy^30^10. Nair^43^ 11. Gupta^42^ 12. Deininger 45 13. Deininger^46^ 14. Gope^48^ 15. Beattie^58^ 16. Bhattacharjee^60^ 17. Arunachalam^53^ 18. Das^54^ 19. Jejeebhoy^49^ 20. Holden^50^ 21. Prillaman^51^RMNCH, reproductive, maternal, newborn and child health. *Three studies had multiple primary outomes with mixed effects: Gupta (11) had main outcomes with positive or no effects. Beattie (15) had main outcomes with positive or no effects. Jejeebhoy (19) had primary outcomes with no or negative effects.

Figure 2 includes three harvest plots for the primary or main health outcomes in moderate-quality and high-quality experimental studies. We made separate plots to describe the relationship between intervention effects and levels of community participation, scope of capability strengthening and underlying group type, as defined in panel 1. We found more studies with positive effects as the level of community participation increased from informing community members (n=2/7) or consulting them (n=1/2) to building a partnership (9/12). Similarly, we found more studies with positive effects when interventions aimed to increase community capabilities (n=7/9) rather than focusing only on building individual (n=1/2) or group capabilities (2/7). Lastly, we found more studies with positive effects through open or community-based groups (n=7/10) compared with SHGs (4/9).

#### Effects by type of social and behaviour change techniques employed

**Figure 3:**
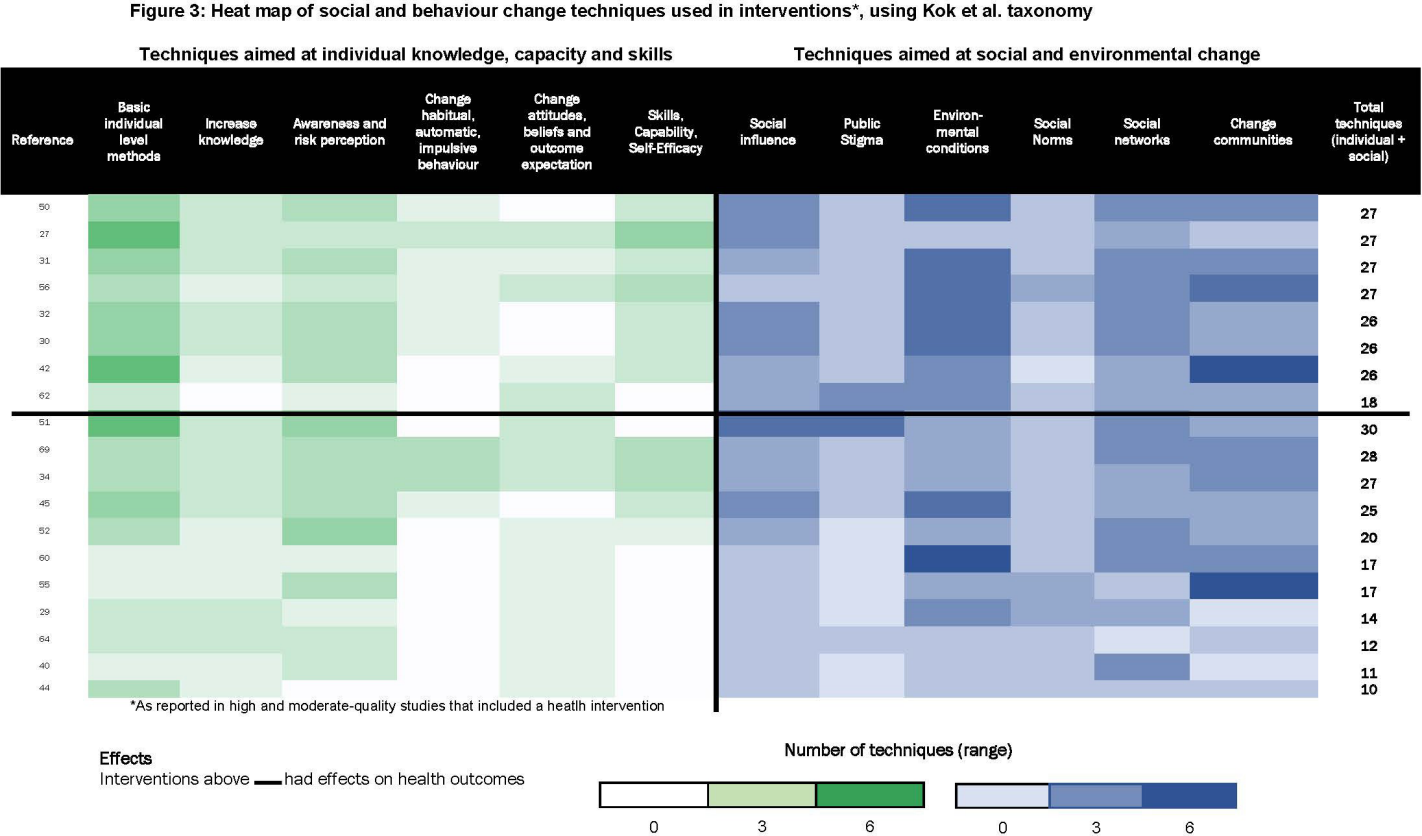
Heat map of social and behaviour change techniques used in interventions

Figure 3 is a heat map of social and behaviour change techniques used in group interventions, using a taxonomy developed by Kok *et al*.^21^ It illustrates two findings. First, on average, interventions that succeeded in improving health outcomes^25 28–30 40 53^ used more social and behavioural change techniques (mean: 25.5, SD: 2.9) than those that did not succeed in improving health outcomes (mean: 19.2, SD: 6.9), with only a few exceptions.^32 43^ Second, successful interventions tended to use a combination of: (A) individual techniques aiming to increase knowledge and risk perception and (B) techniques to foster wider social and environmental change, including techniques to change social norms, and participatory problem posing and solving. Interventions that employed fewer, or mainly individual-level, techniques reported positive effects on self-reported behaviours but not on ‘harder’, objectively measured health outcomes (eg, mortality or anthropometry).^27 42^ In sum, using more and more diverse techniques mattered, especially to achieve changes in ‘hard’ outcomes.

### Enablers and barriers in group-based interventions

- Table 1 summarises enablers and barriers related to context, intervention design, implementation and outcome characteristics.

**Table 1:** Enablers and barriers

**Thematic category**	**Enablers**	**Barriers**
Contextual factors	Presence of existing SHGs	Migration (rural and urban)
	Community willingness to develop groups	Poor supply of health services
	Partnerships with municipalities in urban areas	
Intervention design and implementation	Problem solving to identify feasible solutions that engage women	Giving health messages without women’s active participation
	Trusted, local female facilitator who leverages local practices and beliefs	Poor outreach to target women and influencers
	Inclusion of most vulnerable through active engagement	Group dissolution
	Sufficient coverage to improve population health	Irregular attendance
	Intergenerational participation, such as mothers-in-law and adolescents	Insufficient time spent on health, including duration and frequency
Outcome characteristics	Relevant to majority of group members and local community	Driven by intrahousehold dynamics and social norms
	Supply-independent mechanisms to achieve effects possible or intervention addresses supply	Dependent on diffusion
	Limited, focused outcomes	

SHGs, self-help groups.

#### Context

Two commonly cited contextual barriers to success were the lack of adequate health services in rural areas and high levels of migration in urban areas.^27 28 32 43 49 67 69^ Several quantitative and qualitative studies cited the presence of pre-existing SHGs as a key contextual enabler to improving health. Many hypothesised that SHG membership itself could improve financial security and health behaviours, which in turn would improve health outcomes.^70–73^ However, our review identified no high-quality or moderate-quality experimental studies reporting effects of SHG-only interventions on hard health outcomes such as mortality or anthropometry and only limited effects on self-reported behaviours.^41 45 51 63 66^ Some researchers argued that the social cohesion of SHGs would make add-on health education interventions more effective.^12 74–79^ Yet several empirical studies identified barriers to integrating health interventions into SHGs: limited priority and time for health, exclusion of the most vulnerable and instability of the ‘platform’ due to group dissolution and irregular meetings.^33 42 50 56 80–83^ Finally, some studies argued that women’s groups could support health interventions through partnerships with government to monitor accountability as well as engage and mobilise communities, which appeared feasible in rural and urban settings.^53 84–91^

#### Intervention design and implementation

Groups that improved health outcomes did not aim to ‘nudge’ new behaviours.^92 93^ Rather, they built individual, group and also communities’ capabilities by encouraging participation, problem solving and locally relevant solutions to address direct and underlying determinants of health behaviour.^40 94–98^ Furthermore, the active involvement of community health workers provided a bridge to health systems.^25 27 29 54 62 69 99^ Motivated, trusted facilitators—local women hired with adequate training—enabled effective meetings, ensured inclusion of the most vulnerable and prioritised health.^94 95 99^ Interventions that recruited SHG members as facilitators noted challenges in leadership, communication and technical capacity.^50 80^ However, externally hired SHG community mobilisers who worked across finance, livelihoods and health juggled multiple priorities and gave limited priority to health.^56 68 82^ Training local women or recruiting existing community health workers emerged as the two most promising models to ensure quality facilitation that capitalised on local trust, knowledge and health systems links.^25 29 62 94 99^

Effective group interventions attained sufficient intervention intensity: meetings held at least monthly, ranging from 1 to 2 hours per meeting, and over 1 year or more.^26 30 96^ Others reported irregular participation due to migration or lack of priority, resulting in limited time to discuss health—sometimes as short as 10 min^42^—and inadequate intervention duration to improve health outcomes.^32 42 62 80^ Groups that improved population health outcomes, primarily open groups, attained sufficient coverage of concerned women, for example, pregnant women when groups were concerned with improving RMNCH.^28 29^ Open groups in rural areas reported that over 55% of targeted women attended meetings, whereas a similar intervention that did not achieve effects in urban areas reached only 8% of reproductive-aged women.^28 29 32^ Observational studies reported limited coverage of young mothers in SHGs^100 101^: specific to RMNCH interventions, only one in four mothers with children under 2 years were SHG members in three states.^12^ Stability of groups varied: 27% of original microfinance and health groups in rural Bihar dissolved over a 1-year study period^34^ and open groups in Mumbai had 30% annual population turnover,^32^ while rural, open groups and sex worker collectives sustained participation over longer intervention periods.^59 95^ Lastly, intergenerational participation in groups was noted as important to address culturally rooted practices or household dynamics where mothers-in-law and family play an important role, such as birthing practices or domestic violence.^92 102^

#### Outcome characteristics

Women and community members participated in group activities when topics discussed were relevant to them, such as neonatal practices in high-mortality settings or condom use among sex workers.^32 95 98^ This was key to success: not enough women in urban Mumbai were interested in perinatal practices to sustain continued group participation, possibly because mortality rates were lower in this setting and improvements in birth outcomes depended on the quality of facility-based care, which required other mechanisms to influence.^32^ Government SHG members have a mean age of 38 years,^103^ with typically two to four members who are pregnant or mothers of young children, making the success of RMNCH interventions entirely dependent on diffusion to non-SHG members.^12 34 42 100^ Inclusion of more outcomes to sustain interest among other members did not appear effective: interventions with more than two health domains had limited or no effects, plausibly due to lack of focus.^62 67^ Effective interventions addressed outcomes with mechanisms that were in women’s control or addressed supply-side factors. For example, neonatal survival improved through supply-independent mechanisms such as wrapping newborn infants, while child wasting, stunting and underweight only improved with direct food provision.^48 95^ Similarly, group-based gender sensitisation training was perhaps insufficient to address the patriarchal social norms that perpetuate violence against women.^49–51 102 104 105^

## Discussion

We have conducted the first mixed-methods systematic review of the effects, enablers and barriers to groups improving women’s and children’s health in India, a setting where groups are widely used for health promotion. Experimental studies provided moderate-quality evidence that health interventions with groups can improve perinatal care practices, neonatal survival, immunisation rates, women and children’s dietary diversity and the control of vector-borne diseases. There was stronger evidence for interventions that were relevant to group members, actively built communities’ capabilities, used multiple social and behaviour change techniques and attained sufficient implementation intensity. These characteristics resonate with existing social and behaviour change theory.^21 22^ Our finding that groups need to be engaged through multiple behaviour change techniques beyond those used with individuals also concur with the proposals made in a recent framework for behaviour change through groups and a review of techniques employed in low-income and middle-income settings.^106 107^

Evidence of positive effects on maternal, newborn and child health outcomes among rural, open women’s groups engaged in community mobilisation aligns with findings from global systematic reviews.^2 9 14 108^ The lack of evidence of effects on violence against women and anthropometry underscores the limitation of group interventions when constrained by adverse, deeply rooted social norms or a limited supply of health and nutrition services.^8 97 109^ Like other systematic reviews, we found little evidence that SHGs can improve health outcomes on their own.^6–8 10 110^

In a separate article, we identified three ‘ideal types’ of group interventions to improve health: ‘classrooms’ that build individual capacities using the group as a platform for information dissemination; ‘clubs’ that intentionally build group capacity to address health among members; and ‘collectives’ that engage communities to identify and address underlying determinants of their health problems.^111^ This review found limited evidence that classroom-type interventions are effective beyond improving self-reported knowledge or behaviour among group members.^42 62^ Examples of the club approach noted the importance of investing in group strength and actively facilitating group action for health.^38 53^ Collectives that invested time in participatory approaches more commonly reported improvements in outcomes at a population level.^25 28 29 40^

SHGs are widely viewed as a useful platform to improve health in India, but our synthesis suggests that adding a health education component to meetings is unlikely to change population-level outcomes without opening health interventions up to non-SHG members, using both individual-level and community-level social and behaviour change techniques, and addressing common barriers to intervention intensity, such as giving too little time to discussions about health.^42 50 80^ Our review does however suggest promise for SHGs as community mobilisation partners in broader population health interventions, as illustrated by effective interventions for vector-borne disease control.^53 54^ For group-focused interventions, health issues beyond RMNCH—such as non-communicable diseases and access to entitlements—may be more aligned with the age profile of SHG members.^101 112–114^

Our review has limitations. Many experimental studies included multiple secondary outcomes, but we limited our syntheses to primary or main reported outcomes, which may have led us to under-report effects for intermediate behaviours. We did not examine effects by population subgroups (eg, among the poorest), due to heterogeneity in outcomes and common lack of reporting by subgroup. Many studies did not provide sufficient detail on intervention design and processes, such as meeting frequency, facilitator characteristics or behaviour change approaches, and we did not contact authors for additional information. Furthermore, the Kok *et al* taxonomy was designed to guide intervention development rather than code techniques and thus contained some overlapping categories.^21^

Our recommendations were influenced by limitations in the evidence base. We found few evaluations from urban areas. One-third of experimental studies were at serious or critical risk of bias, largely because evaluations did not adequately address selection bias, missing data or failed to prespecify their main outcomes. Group-level findings that did not report population coverage limited our ability to examine the potential of such interventions to improve population health and equity. Only 13 experimental studies included process evaluations or qualitative findings, limiting the strength of our conclusions on enablers and barriers.^29 32 38 50 51 53 60 62 67 68 80 82 94 95^ Lastly, only 12 evaluations included cost data.^28 30 42 43 45 48 52 55 56 67 115 116^

Box 2 summarises this review’s recommendations for future interventions with women’s groups in India. These have potential relevance for other countries that have community engagement programmes with women’s groups, including Bangladesh, Nepal, Thailand, Bolivia, Haiti, Ethiopia, Nigeria and South Africa.^6^ Future research should estimate population-level coverage of groups and effects, rather than focusing solely on group members. More robust evaluations are needed from urban contexts and for key areas including family planning, water, sanitation and hygiene, non-communicable disease and violence against women. Studies should aim to include objectively measured health outcomes and measures to address social desirability bias with self-reported behaviours. Lastly, systematic reporting of behaviour change approach, group and intervention implementation processes and costs will help to inform policy and practice.^21 117^

Box 2 **Panel 2: Ssuggested principles for women’s groups interventions to improve health**

- Interventions with women’s groups should focus on changing health outcomes that are supply- independent or concurrently address supply-side factors.
- Groups should either open up to those interested in the selected health outcomes, choose health outcomes aligned with members’ needs or allow members to decide which outcomes they wish to focus on.
- Community interventions with women’s groups should go beyond disseminating information and seek to build communities’ capabilities. Short modules to deliver health messages are rarely effective for outcomes that are determined by more than knowledge deficits.
- Women’s groups interventions should have sufficient intensity: most successful interventions have groups that meet at least monthly, for at least an hour focused on health and for an intervention duration proportionate to the complexity of the outcome(s) tackled.
- Group-based interventions should aim to involve the wider community through specific, intentional mechanisms (eg, community meetings or outreach) given that most groups will not attain sufficient population coverage and there is limited evidence of diffusion.
- Motivated and capable facilitators—local women with adequate training or support—are required for almost all social and behaviour change approaches.
- Community health workers should be engaged in women’s group interventions, either as facilitators or as participants, to facilitate links with the health system.

## Conclusion

Community interventions with women’s groups can improve women’s and children health in India if they engage with whole communities and with sufficient intensity. Our review suggests that using women’s groups only as a platform to disseminate health messages has limited rigorous evidence of effectiveness on population-level outcomes. There is more promise in community mobilisation approaches that seek to build communities’ capabilities. These should focus on changing health outcomes that are of interest to group members and are either supply independent or with a concurrent focus on supply-side factors, have sufficient intensity, population coverage and good facilitators, preferably connected to the health system.
