# Supplementary material for: Community interventions with women’s groups to improve women’s and children’s health in India: a mixed-methods systematic review of effects, enablers and barriers
Source: BMJ Glob Health. 2020 Dec 15;5(12):e003304. doi: 10.1136/bmjgh-2020-003304 (PMC7745316; doi:10.1136/bmjgh-2020-003304)

Supplementary Text

Domains with less than three studies

Two studies on health expenditure reported no effects: a high-quality RCT of a health education intervention with a community-based women's group found no effects on women's utilisation of health insurance (aRR: 1.03, 95%CI: 0.81,1.30)[64] and a moderate-quality quasi-experimental rural study of SHG membership found no effects on health care expenses (intervention: USD 840 vs USD: 948 control, p=0.13).[65] Two studies at high risk of bias found positive effects of supply-side inputs on water and sanitation with rural SHGs on water quality (geometric mean thermotoler and coliform count: (13.7 [95% CI: 9.9-18.8; vs 44.5; 95% CI: 33.7-58.8]; p=0.01)[66] and toilet construction (intervention: 48% vs. 15% control).[67] Of studies that targeted multiple health domains, a high-quality RCT of participatory women's groups meetings combined with community resource centres and home visits in urban, informal settlements found improvements in unmet need for family planning, but not on child immunisation or wasting (aOR: 1.31, 95% CI: 1.11 -1.53; aOR 1.30, 95%CI: 0.84, 2.01; aOR: 0.92 (95% CI: 0.75,1.12, respectively).[69] A low-quality RCT reported no effects of a rural intervention to introduce participatory learning and action through SHGs on over 50 health outcomes.[70] Lastly, two moderate-quality studies that measured effects on mental health found no improvement in health-related capability indicators from SHG membership[68] or on overall maternal depression, except in the final year of a rural community mobilisation intervention with open groups (aOR: 0.74 95% CI: 0.40-1.37).[30]

Supplementary Table 1: Search terms

Search domain	Query
**1: Women**	Woman OR Women OR Matern* OR Mother*
**2: Groups**	Group OR Groups OR Club OR Committee* OR Collectiv* OR Meeting* OR Participat* OR Organis* OR Organiz* OR microfinance OR saving OR credit OR insurance
**3: Health**	Health OR illness* OR disease OR disorder* OR infect* OR well-being OR morbidit* OR medical* OR medicine OR deliver* OR Hospital OR Hospitals OR Hospitali?ation OR Child OR Children OR family* OR neonat* OR mortality OR reproductive OR sexual OR HIV OR condom OR family?planning OR contracept* OR sterili* OR mental OR depress* OR anxiety OR stress OR support OR emot* OR violen* OR psychosocial OR malaria OR tuberculosis OR diarrh* OR incidence OR respirator* OR utili* OR service* OR expen* OR insur* OR financ* OR bednet OR water OR toilet*
**4: Nutrition**	Nutrition OR Micronutri* OR Macronutri* OR Body Mass Index OR Anthropometr* OR Arm circumferen* OR Stunt* OR Wast* OR Underweight OR Anemi* OR Hemoglobin OR Diet OR Dietary OR Food OR Feed* OR Calori* OR Grow* OR Breastfe* OR Complementar* OR Feed* OR Birth* OR weigh* OR Vitamin*
**5. Search string**	1 and 2 and (3 or 4) and 5 and 6

Supplementary Table 2: Randomised controlled trials on the effects of women's groups

First author (year of publication)	Setting	Intervention	Group type	Scope of capacity building for health (individual, group, community or none)	Level of participation (informing, consultation or partnership)	Intervention duration	Control	Participants (n intervention, n control)	Level of outcome measurement (group only, members and non-members, population-level)	Primary outcome(s)	Effect size (95% CI)	Risk of bias‡
**Reproductive, maternal, newborn and child health**												
Kumar (2008)*	Rural Uttar Pradesh	NGO-trained, salaried community volunteers facilitated 4 monthly newborn care stakeholder meetings, 3 monthly community meetings, 3 monthly folk song meetings, one monthly volunteers' meeting and did two antenatal and two postnatal home visits to promote birth preparedness, essential newborn care and danger sign recognition.	Open	Community	Partnership	16 months (2004-2005)	Usual care	Pregnant women in the study area identified through a retrospective survey of all women of reproductive age at baseline (2007) and then prospectively until 2010 (Int n infants=1581; Con n=1143)	Population	Neonatal mortality	Adjusted Risk Ratio [ARR]: 0.46 (0.35,0.60)	Some concerns
Kumar (2012)*	Rural Uttar Pradesh	As above	Open	Community	Partnership	As above	Usual care	Pregnant women in the study area identified through a retrospective survey of all women of reproductive age at baseline (2007) and then prospectively until 2010 (Int n	Population	Maternal mortality	ARR: 0.44 (0.14-1.43)	Some concerns

								mothers=2681; Con n=1129)				
Tripathy (2010)*	Rural Jharkhand and Odisha	NGO-trained, salaried local women facilitated monthly women's groups meetings with a mix of newly formed groups and self-help groups opened up to non-members. Meetings followed a Participatory Learning and Action cycle in which group members identified and prioritised common perinatal problems, discussed and implemented strategies to address these, and evaluated their results. Groups organised two community meetings to elicit support for strategies. All clusters received Village Health Nutrition and Sanitation Committee (VHNSC) strengthening.	Mix of SHGs that opened up to non-members and newly formed open groups	Community	Partnership	Three years (2005-2008)	VHNSC strengthening only	All women who gave birth during the study period and their infants, (Infants Int n= 9388; Con= 8819)	Population	Neonatal mortality, maternal depression	AOR for neonatal mortality: 0.68, 95% CI: 0.59-0.78, moderate maternal depression AOR 0.74 (0.40-1.37)	Some concerns
Houweling (2013)*	Rural Jharkhand and Odisha	As above	Mix of SHGs that opened up to non-members and newly formed open groups	Community	Partnership	Three years (2005-2008)	VHNSC strengthening only	Less marginalized (Int n= 4384 Con n=4219) versus most marginalized mothers and infants (illiterate, very	Population	Neonatal mortality	AOR for most marginalised 0.41 (0.28; 0.59) AOR for less marginalized 0.64 (0.51; 0.80)	Some concerns

								poor, with little or no land, and belonging to Scheduled Tribes or Scheduled Castes (Int n= 1897 Con n=1612)				
Acharya (2015)*	Rural Uttar Pradesh	NGO-trained, incentivised ASHAs facilitated newly formed monthly mothers' group meetings using oral and pictorial participatory methods to promote birth preparedness, essential newborn care and danger sign recognition. They were supported by Village Health Sanitation and Nutrition Committees and mass 'mid' media campaigns.	Community-based women's group	Group	Informing	Three years (2007-2010)	District-level campaigns with advocacy, mass media and "mid-media" (e.g., local street theatre)	All women who gave birth during between 2007 and 2010 in the study clusters (Endline Int n infants=5988; Con n=5897)	Population	Neonatal mortality	AOR: 0.98 (0.80,1.19)	Some concerns
More (2012)*	Mumbai, Maharashtra (informal settlements)	NGO-trained, salaried local women facilitated newly formed fortnightly women's groups meetings in informal settlements. Women improved their knowledge of local perinatal services, best practices and how to negotiate optimal care with family and	Open	Community	Partnership	Three years (2006-2009)	Usual care	Women who gave birth in the study clusters and their infants (Infants, Int n=7656; Con n=7536)	Population	Stillbirth rate, Neonatal mortality rate	Stillbirth rate AOR: 0.66 (0.46,0.93), Neonatal Mortality AOR 1.42 (0.99-2.04)	Some concerns

		providers through peer-learning and the identification and implementation of local strategies following Participatory Learning and Action and Appreciative Inquiry approaches.										
Tripathy (2016)*	Rural Jharkhand and Odisha	NGO-trained, incentivised ASHAs facilitated monthly women's groups meetings with a mix of newly formed groups and self-help groups opened up to non-members in their own working areas. Meetings followed the Participatory Learning and Action cycle described above. All clusters also received Village Health Nutrition and Sanitation Committee (VHNSC) strengthening.	Open	Community	Partnership	Two years (2011-2012)	VHNSC strengthening only	All women who gave birth during the study period and their infants, (Infants Int n= 9388; Cont= 8819)	Population	Neonatal mortality	AOR 0.69 (0.53, 0.89)	Low
**Nutrition**												
Nair (2017)*	Rural Jharkhand and Odisha	NGO-trained, salaried community health workers did monthly home visits in the third	Open	Community	Partnership	30 months	VHNSC strengthening only	Pregnant women identified and recruited in the study clusters and their children	Population	Children's length-for-age z score at 18 months	Adjusted mean difference 0.11 (–0·01, 0·23)	Low

		trimester of pregnancy and the first 24 months after birth, and facilitated a cycle of Participatory Learning and Action meetings focused on identifying problems and strategies related to maternal and child health and nutrition in the first 1000 days of life. All clusters also received VHNSC strengthening.						(Infants Int n=1460; Cont n=1541)				
Gupta (2019)*	Rural Bihar	Jeevika community mobilisers met with SHG members (women aged above 18 years who participate in microfinance activities) to deliver messages on maternal and child health, nutrition and WASH twice a month through videos on health and nutrition, as well as targeted home visits, peer group meetings, and community events.	SHG	Individual	Informing	2.5 years (2016-2018)	Groups with no health and nutrition intervention	Women belonging to a household where at least one woman was a member of a Jeevika SHG and with at least one child aged 6–23 months (Endline households n=2119)	Group	Women's BMI<br><br>Dietary diversity for children aged 6–23 months	Effect on mean BMI z score:<br>B coefficient: -0.025 SE: (0.082)<br><br>Reported dietary diversity of youngest child:<br>B coefficient: 0.286 SE (0.118)<br><br>Reported dietary diversity of index child:<br>B coefficient: 0.169 SE (0.080)	Low
Ojha (2019)*	Rural Bihar	NGO-supported women's self-help groups (SHGs) involved in	SHG	None	Informing	18 months	Usual care	All children < 5 years resident in study clusters (n= 2534 in total)	Population	Weight-for-height z score of children < 5 years	AOR: 0.46 (0.28, 0.74)	High

		savings and credit activities through four weekly SHG meetings and after six months, loans from the NGO for emergencies (e.g. access to health services) and general purpose (e.g. investment and consumption purposes).										
**Violence against women**												
Jejeebhoy (2017)*	Rural Bihar	Peer facilitators met fortnightly with SHGs supported by the Women Development Corporation (WDC), and monthly with husbands of SHG members. The intervention included gender transformative group learning sessions with SHG members and similar sessions with husbands; activities to link SHG members with livelihood training opportunities; and community mobilisation at the village level by SHG members and their husbands to change gender norms and attitudes.	SHG	Group	Informing	15 months (2014-2015)	Groups with usual care	Married women in SHGs aged 18-49 years residing in the study areas Endline: Arm I (n=567) Arm II (n=531 members); n=1053 non-members Arm III (n=588 members) (n=1025 non-members)	Members and non-members	Attitudes relating to gender roles among SHG members<br><br>Experience of marital violence	Index of gender role attitudes AOR: 0.69 (0.35, 1.02)<br><br>Violence: Emotional AOR: 2.95 (1.75,4.97) Physical AOR: 0.69 (0.46,1.02)<br><br>Sexual AOR: 1.23 (0.64,2.36)	Some concerns
Holden (2016)*	Urban Madhya Pradesh	Trained facilitators delivered	SHG	Group	Informing	15 months (2013-2014)	Usual care	SHG members (Endline n=1751) and	Members and non-members	Women: 1. Experience	Women's experience of physical or	Some concerns

	(informal settlements)	training to: strengthen SHG functioning and gender training with information on Violence Against Women and Girls (VAWG) referral networks; increase women SHG members' understanding of the root causes and trigger factors related to VAWG and build women's capacity to take action and respond to VAWG. This included meetings to connect the SHGs and their members with services to prevent and respond to VAWG, and SHG members doing women's safety audits to identify actions that might be taken to improve the safety of slum areas. Life skills training with men and boys through new groups of adolescent boys and young men (aged 15-25 years) to build their capacity to challenge harmful social norms and take actions against						adult women 18-49 in the same community who were not members (Endline n=1660)<br><br>Men and boys (older and younger) in the community, direct and indirect beneficiaries		of physical and/or sexual IPV 2. Experience of violence and harassment in public spaces Men: perpetration of physical and/or sexual IPV and perpetration of violence and harassment against women and girls in public spaces.	sexual violence: Female direct Coefficient (SE) -0.006 (0.022) Female indirect 0.002 (0.021) Perpetration of physical or sexual violence by men 0.025 (0.075) Male indirect -0.003 (0.017) Experience of violence or harassment in public spaces Female Direct 0.005 (0.017) Female indirect 0.03* (0.017) Male Direct (perpetrated) 0.022 (0.02) Male indirect 0.018 (0.015)	

		VAWG in the community.										
**Vector-borne diseases**												
Arunachalam (2012)*	Peri-urban Tamil Nadu	10 SHGs were enrolled, and one person from each SHG was identified as a 'dengue' focal point to mobilize other members of the group. The community participated in the distribution of water container covers and health education materials and helped researchers to organize meetings. School heads in the intervention clusters were informed about the objectives of the dengue project and encouraged the teachers and students to participate,. Educational materials were distributed through SHGs and school children in the intervention clusters. Netted frames of three sizes were made locally by sub-contractors and distributed to each household with an accompanying SHG member.	SHG	Group	Consultation	c.10 months (2009-2010)	Usual care	Households in study areas (Int households n=1000 Con= 1000)	Population	Mosquito pupal indices in house and per person	Difference in reduction, between two arms<br><br>House index for pupae: Difference: -14.7%, p=0.012<br><br>Per person pupae index: Difference: -0.35, p=0.0200	Some concerns
Das (2014)*	Rural	Two	SHG	Community	Partnership	c.12-15	Group with	All individual	Population	Unclear, but	- Total %	Some

	Odisha	interventions were tested. In Arm 1, NGO field workers visited ASHAs at least twice a month to share information about the transmission, diagnosis and treatment of malaria; hands-on support for performing and interpreting rapid diagnostic tests; administration of the correct dosage of ACT and follow-up to ensure compliance; management of malaria surveillance records; orientation on community mobilisation and health centre engagement. Community mobilisation focused on increasing consistent use of insecticide treated bed nets provided to the community free of cost by government, and timely care-seeking for febrile illnesses from the ASHA. Messages were disseminated to local governance bodies, social organizations, women, men,				months (2010-2011)	usual case management by ASHA, with no other support	recent fever cases within each village in study areas, n= 768 (Arm 1); n=781 (Arm 2); ; n=755 (Arm 3, Control)		possibly: (1) % HH who used at least one LLIN 2) % HH who had fever tested for p. falciparum in 24 hours	population slept under bed net last night: Arm B (community mobilisation) vs Control: 1.274 (1.143, 1.419)<br><br>- Fever diagnosis <24 hours Total pop, B vs control: 1.01 [0.74,1.38]<br><br>-Prompt diagnosis by trained provider: B vs control: 1.45 [1.086,1.937]	concerns

		youth, school and religious groups were chosen through posters, leaflets, cinema shows and street plays. SHGs were assigned 10-15 homes each in every village to monitor bed net use at night. Arm 2 included community mobilisation alone, without training for ASHAs. .										
**Sexual health and HIV**												
Sherman (2010)†	Peri-urban Tamil Nadu	Health educators offered sex workers eight hours of HIV prevention education through didactic methods and interactive activities twice weekly, plus 100 hours of tailoring training taught by master tailors.	Special population group	Individual	Informing	Five weeks	HIV prevention education only	Sex workers aged over 18 years (Int n=58 Con = 48)	Group	Mean n of sex exchange partners	Int mean: 3.1 [2] Con mean: 5.1 [3] p <.0001	High
Spielberg (2013)†	Rural West Bengal	An NGO trained local self-help promoting institutions to deliver learning games for Girls and other non-formal education on health, livelihoods, and family finance to SHGs of poor women and adolescent girls during their regular savings and	Special population group	Group	Informing	Three years (2006-2009)	Usual care	Women SHG members who attended the first session (Int n=471, Con n=409), and their daughters or daughters in law (Int n= 897, Con n= 450)	Group	% who ever heard of HIV	AOR: 3.6 (1.6, 8.0)	High

		loan meetings. Health education included information on diarrhoea, hygiene, and HIV to SHG members and their daughters or daughters in law, and to SHGs with adolescent girls only.										
**Health expenditure**												
Desai (2017)*	Peri-urban Gujarat	NGO-trained health workers provided preventive care group health education to women on hysterectomy, diarrhoea, fever/malaria and sanitation using films, interactive discussions and games.<br><br>All clusters received home visits and group education on common illnesses (excluding diarrhoea, malaria and hysterectomy), accompaniment to referral to health services; medicine sales and insurance promotion; linkages with government providers; support to activate VHSNCs.	Community-based women's group	Individual	Consultation	18 months (2010-2011)	Regular SEWA community health worker services	For primary outcome: all women who had made an insurance claim in the study clusters; for secondary outcomes: insured and uninsured women in study clusters at baseline and follow-up surveys. Participants were 3340 insured women residents, point, Int n=1436 person–years; Con n=1227 person–years.	Population	Insurance claims rate for 3 conditions (malaria, hysterectomy and diarrhoea)	ARR: 1.03; 95% CI: 0.81, 1.30	Low
**Multiple outcome domains**												
More (2017)*	Mumbai,	Community	Open	Community	Partnership	Three years	No Centre	Ever-married	Population	Met need for	Met need for	Low

	Maharashtra (informal settlements)	Resource Centres were created and employed full-time, salaried community organisers who made home visits, organised group meetings, provided services, day care for malnourished children, did community events and liaised with existing systems.				(2011-2014)		women aged 15-49 years residing in study clusters and any children under five years		family planning, full immunisation for children, childhood wasting	family planning, AOR:1.31, (1.11, 1.53). Full immunisation for children : AOR 1.30, (0.84, 2.01) Childhood wasting: AOR 0.92, (0.75, 1.12)	
Subramanyam (2017)*	Rural Bihar	Jeevika community mobilisers facilitated fortnightly meetings with self-help groups that opened up to non-members, using a Participatory Learning and Action cycle focusing on identifying and addressing problems related to maternal and child health and nutrition, WASH and violence against women, with two wider community meetings.	SHG	Community	Consultation	12 months (2015-2016)	Usual care	Household heads and women of reproductive age, pregnant women and their husbands, community mobilisers and Anganwadi workers (AWW) in study areas: at endline, n=3340 households, n=1612 women, n= 282 community mobilisers n=233 Anganwadi workers	Population	59 outcomes related to health, nutrition, violence and social capital – no primary outcome specified	The authors found no evidence of effects on health, nutrition, WASH and violence.	High

* Cluster randomised controlled trial † Individual randomised controlled trial ‡ Risk of Bias assessment based on Cochrane ROB-2 [18]

Supplementary Table 3: Quasi-experimental studies on the effects of interventions with women's groups

First author (year of publication)	Setting	Intervention	Group type	Scope of capacity building	Level of participation	Intervention duration	Control	Study design	Participants (n intervention, n control)	Main outcomes *	Population or group-level measurement	Effects (95% CI or SE)	Risk of bias†
**Reproductive, maternal, newborn and child health**													
Roy (2013)	Rural Jharkhand and Odisha	The Participatory Learning and Action cycle tested in Tripathy (2010), implemented in the previous RCT's control areas	Open	Community	Partnership	Three years (2009-2011)	No control for implementation in control areas	Non-randomised, controlled	All women who gave birth during the study period and their infants, (Infants n=39,918)	Neonatal mortality	Population	AOR:0.69 (0.57–0.83)	Moderate
Saggurti (2018)	Rural Bihar	Community health facilitators did eight weekly cycles of participatory communication (banners, stories, picture cards or songs with messages) with existing women's self-help groups using different thematic modules on antenatal and postnatal care, maternal and child nutrition, routine immunisation, family planning, personal hygiene and use of toilet.	SHG	Individual	Informing	12 months (2013-2014)	SHGs with no health intervention, no matching	Non-randomised, controlled	Group members who had a birth in the past 12 months (Endline Int women n=718; con = 217)	Maternal, neonatal and child health knowledge and practices	Group	Adjusted Difference In Difference [ADID]: 4+ antenatal care visits: -0.4 (-6.2, 5.5); Consumption of IFA tablets/syrup for 100+ days: 4.9 (-1.1, 10.8); Institutional delivery: 8.8 (-0.1, 17.8); visit by a health worker within 2 days after delivery: -4.6 (-13.6, 4.4); Skin-to-skin care for newborn infants: 17.0 (-0.5, 34.1); Delayed infant bathing for 3+ days: 19.2 (3.8, 34.6); Timely initiation of breastfeeding: 20.5 (5.7, 35.3); Exclusive breastfeeding: 26.7 (9.4, 44.1); Fed solid/semi-solid food: 4.7 (-5.3, 14.6); Age appropriate immunization: 9.1 (1.0, 19.6); Use of modern contraception methods: 9.3 (1.3, 17.2)	Serious

Saha (2015)	Rural Gujarat and Karnataka	Facilitators from two NGOs (Self Employed Women's Association, or SEWA and Shri Kshetra Dharamstala Rural Development Project (SKDRDP). SEWA included health insurance and primary health care delivered through stationary and mobile health camps, health education and training, the production and marketing of traditional medicines. SKDRDP included health education in routine credit group meetings, home visits by a village health worker, the promotion of low latrines, and insurance with health cover.	Community-based women's group	Individual	Consultation	One year (2012-2013)	Groups with no health intervention, matching at block and group level	Non-randomised, controlled	Women of reproductive age with a child younger than two years (Women, Int = 219; Con = 253)	Institutional delivery; Feeding a newborn infant colostrum; Having a toilet at home; Diarrhoea among children	Group	Institutional delivery AOR 5.08 (1.21-21.35)<br><br>Feeding colostrum AOR 2.38 (1.02-5.57)<br><br>Have a toilet at home AOR 1.53 (0.76-3.09)<br><br>Diarrhoea among children AROL 0.86 (0.42-1.76)	Moderate
Prennushi (2014)	Rural Andhra Pradesh	Government-employed community resource persons formed SHGs. Government gave SHGs seed funds and links to banks to expand access to low-cost credit and training in social and economic skills. Government also set up federations of SHGs in villages, blocks, and districts.	SHG	None	Informing	Four years (2004-2008)	Usual care	Propensity score matching with panel survey	4,250 households	Assisted delivery<br>Breastfeeding<br>Immunization<br>Knowledge of diarrhea treatment<br>Knowledge of FP methods<br>Visit by FP worker	Group	DID % points:<br>Assisted delivery: +2 %<br>Breastfeeding: + 3%<br>Immunization: -11%<br>Knowledge of diarrhea treatment: +2%<br>Knowledge of FP methods: +2%<br>Visit by FP worker: +2%<br>*None significant at 0.05 level	Moderate
Hazra (2019)	Rural Uttar Pradesh	Swasthya Sakhi, SHG members, were trained as volunteer peer educators. They conducted monthly meetings with SHGs where she disseminated maternal and child health	SHG	Group	Informing	24 months (2015-2017)	SHGs with no intervention, block-level matching	Non-randomised, controlled	Eligible women from SHG household were currently married, 15-49 years and had given birth in the 12 months	1.Reproductive and maternal health practices: At least four ANC vis- its, at least three	Group	Net change, %, with CI<br>At least 4 ANC visits 5. 2 [1.6, 8.7]<br>At least 3 tests during ANC visits: 8.3 [4.4, 12.2]<br>Consumption of 100 + IFA tablets during	Serious

		information on preventive and care-seeking perinatal care practices and family planning. Community outreach activities including home visits, community meetings using of audio visual aids such as health videos.							prior to the survey, Intervention: n=2165; Control: n=2085 d	ANC check-ups, consumption of 100 or more iron folic acid (IFA) tablets, institutional delivery, PNC check-up within first seven days of delivery, and current use of any contraceptive method. 2. Newborn care practices Clean cord-care to prevent cord infection, skin-to-skin care to keep the newborn warm, timely initiation of breastfeeding, and exclusive breastfeeding.		pregnancy 1. 9 [−0.9, 4. 8] Institutional delivery -0.7 [−3.7, 2.3] Postnatal check-up within a week of delivery 4.6 [1.0, 8.2] Current use of any contraceptive method 11.2 [7.0, 15.4], Clean cord care (0–5 months) 7.4 [2.3, 12.4] Skin-to-skin care (0–5 months) 3.7 [−1.6, 9.0] Timely initiation of breastfeeding (0–5 months) 5 .8 [0.1,11.5] Exclusive breastfeeding (0–5 months) -1 .8 [−11.1, 7.4]	
Janssens (2011)*	Rural Madhya Pradesh	The Mahila Samakhya programme set up women's groups in villages. Programmed facilitators women to join the groups and improve their daily lives through collective action, without prescribing the	Community-based women's group	Community	Partnership	5-10 years (data collection in 2003)	Villages with no Mahila Samakhya groups	Regression discontinuity using a single cross-sectional survey	Women who were participants in MS (n=718) and non-participants (n=714) women in control villages (n=559)	Childhood immunization rates	Population	Probit: Programme participant DPT: 0.088 0.037** Measles: 0.076 0.038** TB: 0.071 0.038* (adjusted) Program village DPT 0.195 0.114*; Measles: 0.324 0.103*;	Moderate

		activities that a group have to engage in but assisting women in identifying their own needs and solutions. Groups took up literacy trainings, set up savings and credit groups and informal primary schools for girls. Almost all groups sought to improve their knowledge of health and hygiene through regular visits from a facilitator and health trainings for the groups. Groups conducted their own immunisation campaigns within villages as one of their collective actions.										TB: 0.224 0.131*	
Madhivanan (2013)	Rural Karnataka	SCIL (Saving Children and Improving Lives) delivered integrated antenatal care and HIV testing services to rural villages using mobile medical clinics. In a more intensive arm (SCIL+), a cash transfer was given to local women's SHGs for assisting in mobilizing attendance at the mobile medical clinics. The entire group earned cash that could then be loaned to members.	SHG	Group	Informing	12 months (2011-2012)	Provision of the mobile clinic service without the cash transfer to SHGs	Non-randomised controlled, no adjustments for potential confounders	Pregnant women aged 18 years or more and residing in a study village for more than six months.<br><br>SCIL: 418 pregnancies; SCIL+: 512 p[1]regnancies	Proportion of total pregnancies in these villages for which women received ANC and HIV testing<br><br>HIV prevalence	Population	ANC visit- at least one SCIL clinic attended- SCIL arm- 43% SCIL+ arm received 67% more pregnant women received ANC and HIV testing<br><br>SCIL+ arm- 72.5%<br><br>Prevalence of HIV: SCIL arm- 0.6%; SCIL+ arm-0.9%	Critical
Mozumdar (2018)	Rural Uttar Pradesh	NGO-established SHGs to provide information on healthy maternal and newborn practices by engaging SHG members in discussions on HBMNC (home based	SHG	Individual	Informing	4 months	Households with SHG member in areas without	Non-randomised controlled	Households with at least one SHG member and at least one married woman	Knowledge of maternal and newborn care	Group	DiD results on knowledge (%): Importance of ANC, 10.7*<br>At least 4 ANC check-ups 1.3 2<br>TT injections required, 15.0*	Serious

		maternal and newborn care) topics for one or two meetings per month. SHG members were encouraged to share information on maternal and newborn caregiving with others					health intervention		aged 18-49 years Endline (n=470)			Minimum 100 IFA tablets need to be consumed, 17.4*<br>First PNC check-up for mother within 24 h, 12.7*<br>Number of danger signs during pregnancy, Mean (SD) 1.1*<br>First PNC check-up for child within 24 h, 10.5<br>Number of at least 3 PNC check-ups within 7 days, 4.3<br>Number of danger sign of newborn, Mean (SD1.1*<br>Early BF 6.4<br>Nothing should be applied on cord, 15.2*<br>Keep cord clean and dry, 13.9<br>Delayed bath greater than 48 h, 5.9<br>Heard of KMC, 30.8*<br>Correct method of KMC, 21.4*<br>Return of fertility after 6 weeks 6.5	
Saggurti (2019a)	Rural Bihar	Two modules on newborn health practices and was delivered across 1–2 months in all the groups. Information included immediate postnatal behaviors and breastfeeding practices. Information repeated in the month of implementation (in 3/4 other meetings) and in year 2 but not 3.	SHG	Individual	Informing	2-3 months per module (over three years)	Govt-nurtured SHGs with no health intervention, no matching	Non-randomised, controlled	Married SHG women aged 18-49 with child <6 moths age<br><br>Control:<br>2013, N=112<br>2014, N=183<br>2016, N=99<br><br>Intervention:<br>2013, N=343<br>2014, N=534<br>2016, N=604	Clean cord care; initiation of skin to skin care; timely initiation of breastfeeding; exclusive breastfeeding on day 1; delayed bathing	Group	Intervention vs Control:<br>Clean cord care: 1.9 (1.5–2.3)<br>Initiation of skin-to-skin care: 1.8 (1.5–2.3)<br>Timely initiation of breastfeeding: 1.3 (1.0–1.7)<br>Exclusive breastfeeding on day 1: 1.9 (1.4–2.6)<br>Delayed bathing: 2.3 (1.8–2.9)	Serious

Saggurti (2019b)	Rural Bihar	Eight weekly cycles of participatory behavior communication using different thematic modules on maternal, neonatal, child health and promoting collectivization processes facilitated by community health facilitators or sahelis. The intervention was delivered by sahelis, active women with some literacy and mobility who could learn and deliver health messages.	SHG	Individual	Informing	8 weeks	Group with no intervention, no matching	Non-randomised, controlled	N of eligible women interviewed were: 2407 (in 2013), and 2970 (in 2016) Control: 601 607 Intervention 1806 2363<br><br>Groups interviewed Control: 174, 347 Intervention: 535 1115	(1) whether in the past six months, respondent negotiated with staff of health care provider in order to help a fellow community member (self-advocacy with health care providers), (2) whether in the past six months, respondent negotiated with frontline health workers in villages in order to help a fellow community member (self-advocacy with local frontline workers), and (3) how confident are you in going to a government health center to get reproductive health services	Group	DiD (%)<br>Group-based questions<br>Collective interaction with health facility 15·5 (10·3-20·8), p<0·001<br>Collective agency to negotiate with health centre 1·1 (-3·4-5·7), p=0·626<br>Collective agency to negotiate with anganwadi worker 4·8 (-4·9-14·4), p=0·334<br><br>Individual questions<br>Self-advocacy with health care providers 4·8 (1·8-7·8), p=0·002<br>Self-advocacy with local frontline workers 1.9 (-2·2-5·9), p=0·371<br>Self-confidence in accessing health services 20·3 (12·0-28·7), p<0·001<br><br>Treated fairly by:<br>Accredited Social Health Activists (ASHAs) 1·6 (-3·1-6·3), p=0·512<br>Anganwadi Workers (AWWs) 3·6 (-1·7-8·8), p=0·184<br>Auxiliary Nurse Midwives (ANMs) 1·8 (-2·8-6·4), p=0·438<br>Women reported that ASHAs from health system<br>Treat with respect 3·1 (0·2-6·1), p=0·035<br>Direct to appropriate health providers 6·2 (1·6-10·8), p=0·009<br>Respond quickly to emergency situations 9·7 (3·0-16·5), p=0·005	Serious

										(self-confident in accessing health services).		Available when needed 11·4 (4·8-18·1), p=0·001<br><br>Service availability Iron Folic Acid tablets for 100 or more days 5·3 (3·2-7·4), p<0·001<br>Post-natal care within one week of delivery 14·1 (4·6-23·6), p=0·004<br>Advise on use of clean cloth for drying baby 25·8 (17·2-34·3), p<0·001<br>Advise on use of clean blade to cut the cord 32·4 (23·7-41·1), p<0·001<br>Advise on use of disposable delivery kit 41·1 (33·7-48·6), p<0·001<br>Accompanied by frontline health worker for delivery 14·0 (4·9-23·2), p=0·003	
**Nutrition**													
Gope* (2019)	Rural Jharkhand and Odisha	Two interventions were tested: either NGO-trained and salaried facilitators facilitating a cycle of monthly Participatory Learning and Action meetings focusing on maternal and child health and nutrition home visits to children identified as undernourished using MUAC or Anganwadi records plus creches for children aged 6 months to 3 years;	Open	Community	Partnership	Three years (2012-2015)	Usual care	Non-randomised, controlled	All mothers residing in the study areas who have children aged <36 months (Intervention a n=1256, Intervention b n=1177, Con n= 1130)	% of children under three years who are wasted	Population	Arm a: PLA group + home visits:<br>AOR: 0.66 (0.51, 0.88)<br><br>Arm b: PLA groups + creches:<br>AOR: 0.73 (0.55, 0.97)	Moderate

		or PLA meetings and home visits only.											
De (2011)	Rural West Bengal	Self-help groups with microfinance promoted by the government or through NGO linkages with Govt SHGs for at least 1 year or for 8 years.	SHG	None	Informing	1-8 years	Three controls: 1. Male borrowers 2. Female and male borrowers in SHG for at most 1 year 3. Non-SHG members who wanted to join SHGs	Cross-sectional survey with propensity score matching	Households with female and male borrowers from SHGs int n= 120<br>Con group 1 n= 40<br>Con group 2 n= 90<br>Con group 3 n= 120	Weight-for-age z score among children <15 years<br><br>Household protein intake	Group	Z score/weight for age: coefficient for intervention 0.250, p=0.13 (control 1&2)<br>Z score/weight for age: 0.228, p=0.254 (control 1,2,3)<br>Protein intake: 0.364, p=0.384 (control 1&2)<br>Protein intake: 0.494, p=0.213 (control 1&2)	Serious
Deininger (2012)	Rural Andhra Pradesh	The Society for the Elimination of Rural Poverty (SERP), established by the government of AP, trained facilitators and established federations of SHGs at village, block, district, and eventually state levels, with a focus on including the poorest.	SHG	None	Informing	5 years (2001-2006)	Households with members who have participated in SHGs for 2.5 years	Non-randomised, controlled using propensity score matching	Participant HH involved with SHGs for at least 3 years (n=438); less than 3 years (n=234)<br><br>HH that did not participate in SHGs in treatment villages (n=892) and HH in control villages (n=241)	Consumption (food and non-food items in past 30 days and lumpy items in past year)<br><br>energy intake (Kcal/per day)<br><br>asset accumulation (consumer durables, productive	Population	DiD (SE)<br><br>Energy intake p.c. (kcal/day) 202 (94) p<0.05<br><br>Protein intake p.c. (g/day) 4.35 (2.39) $p \le 0.10$	Moderate

										assets, and livestock assets)			
Deininger (2013)	Rural Andhra Pradesh	The Indira Kranti Pratham (IKP) was implemented in 2 phases, DPIP- District Poverty Initiatives project; RPRP- Rural poverty Reduction Project):<br>i) the program establishes federations of SHGs at village mandal (block), district, and eventually state levels<br>ii) to reach out to the poor.	SHG	None	Informing	3 years (survey conducted in 2004)	House holds in the RPRP areas	Cross-sectional survey with propensity score matching	Int: Households in DPIP areas N=1964; Con: Households in RPRP areas N=3789	Female empowerment; nutritional intake; per capita income, consumption and assets.	Population	Gain in intervention arm, 2001 vs. 2004<br>Energy intake p.c. (kcal/day), 109, $p \leq 0.05$<br>Protein intake p.c. (g/day), 5.84 $p \leq 0.01$	Moderate
**Violence against women**													
Yaron (2018)	Rural Bihar, Uttar Pradesh and Madhya Pradesh	As in Ojha et al (2019)	SHG	None	Informing	18 months	Women in areas with no SHGs	Panel survey with propensity score matching	SHG members in intervention areas and matched adult women in control areas Intervention (n= 740) Control (n=308)	Index of domestic violence	Population	DID:<br>Index of domestic violence mean score: -- 0.448<br>p=0.008	Serious
Prillaman (2017)	Rural Madhya Pradesh	Self-help groups supported by the NGO Pradan.for c.15 years	SHG	None	Consultation	c.15 years	Women in areas with no SHGs	Geographic regression discontinuity design	Women who were part of Pradan SHGs and women in control areas (n=2152 across both areas)	Intimate partner violence	Group	No effects on IPV: -0.092 (0.074) index of violence	Moderate
**Sexual health and HIV**													
Beattie (2014)	Urban Karnataka	Drop-in centres that provided presumptive treatment for Gonorrhea and Chlamydia and meeting place for FSWs to share experiences and gain a sense of solidarity. The program worked to support and develop critical thinking among	Special population group	Community	Partnership	7 years of intervention (2004-2011) evaluated in last three years (2008-2011)	Regression analysis comparing groups with different levels of	FSW not exposed to community mobilization activities	FSWs who sold sex at home, brothels and phone-based in the project area with low, medium medium (attended nongovernmental organization meeting or	HIV and STI prevalence; HIV risk behaviours; and collective and individual power among FSWs	Population	Adjusted analyses: 1st: No/Low vs Med; 2nd: No/Lo vs High (All AOR)<br><br>Visited STI clinic in past 6 months:<br>12.2 (7.89, 18.94)<br>24.5 (15.3,39.3)<br><br>HIV-1 infection<br>1.26 (0.63,2.52)	Moderate

		the FSW community, providing a forum where FSWs could discuss difficulties and reflect on how they could work together to address the challenges they faced through collective action. FSWs formed community-based institutions including peer groups and collectives.					exposure to community mobilisation		drop-in centre) or high (member of collective or peer group) exposure to community mobilisation activities (Endline n= 1934)			1.07 (0.54,2.14)<br><br>Reactive syphilis<br>1.29 (0.47,3.55)<br>0.63 (0.22,1.78)<br><br>HSV-2<br>0.93 (0.44,1.93)<br>0.49 (0.23,1.02)<br><br>Chlamydia<br>0.76 (0.45,1.27)<br>0.64 (0.37,1.09)<br><br>Gonorrhoea<br>0.95 (0.41,2.22)<br>0.39 (0.13,1.19)<br><br>Ever taken HIV test<br>8.15 (4.78,13.88);<br>25.13 (13.07,48.34)<br><br>Condom use last sex, occasional clients:<br>2.28 (1.11,4.69)<br>4.74 (2.17,10.37)<br><br>Condom use last sex, repeat clients<br>2.63 (1.40,4.93)<br>4.29 (2.24,8.20)<br><br>Condom use last sex regular partner<br>1.67 (0.87,3.17); 2.80 (1.43,5.45)	
Bhattacharjee (2013)	Rural and urban Karnataka	Female Sex Workers joined community based mobilization activities including peer groups focusing on building individual capabilities to foster positive perception of self, enhance self-confidence and agency among individual FSWs and promote collective	Special population group	Community	Partnership	Five years (2005-2010)	Usual care	Propensity score matching using 3 surveys	Sex workers taking part in Behavioural tracking survey (BTS) and Integrated Biological and behavioural assessment (IBBA) BTS: Int n=1330 Con n=409	Condom use with regular partner at last sex; Consistent condom use with all partner/clients; Experienced violence	Population	All AOR:<br><br>Gonorrhoea and/or Chlamydia<br>AOR: 0.60 (0.47, 0.78)<br><br>Syphilis<br>0.74 (0.58, 0.94)<br><br>Condom use with regular partner at last sex:<br>1.25 (0.93, 1.68)	Moderate

		identity to address their immediate needs. It also aimed to create an enabling environment by sensitizing a range of stakeholders in and beyond the community level to address factors in the macro-level social environment that creates structural barriers to empowerment among FSWs.							IBBA: Int n=17, Con, n=2937,	in the past six months; Beaten/forced to have sex in past one year; Did not give bribe to police to avoid trouble; Obtained any form of identification document in past five years; Gonorrhoea and/or Chlamydia infection; Syphilis infection; HIV infection		Consistent condom use with all partner/clients 1.07 (0.71, 1.62)<br><br>Experienced violence in the past six months 0.70 (0.53, 0.92)<br>Beaten/forced to have sex in past one year 0.84 (0.62, 1.14)<br><br>Did not give bribe to police to avoid trouble 1.46 (1.04, 2.06)<br><br>Obtained any form of identification document in past five years 1.23 (0.96, 1.58)<br><br>HIV infection 0.89 (0.74, 1.07)	
Shankar (2019)	Urban Maharashtra	A group-based workshop over the course of 8 days, with sessions running 3.5 h/ day. Iindividuals engaged in an introspective examination of aspects of their lives, using a cognitive reframing process, with counselling during and after the workshop.	Special population group	Individual	Informing	8 days	Sex workers who did not participate in the workshop	Individual, non-randomised controlled	Sex workers who participated in full training and one year follow-up (n=58) and controls (n=43)	Adherence to HIV meds<br>Alcohol/tobacco use<br>General health status<br>Desire to leave sex work<br>Left sex work	Group	Increased adherence to HIV meds: 54% vs 0%<br>Decreased adherence to HIV meds: 4% vs 20%<br>Improvement in addiction to alcohol/tobacco: 9% vs 12%<br>Improvement in health status: 52% vs. 19%<br>Deterioration in health status: 2% vs 14%<br>Stated desire to leave sex work: 47% vs 47%<br>Stated desire to leave and left sex work: 34% vs 2%<br>Left sex work: 24% vs 2%	Serious
Swendeman (2009)	Peri-urban	Sonagachi Health Intervention Project activities primarily aim	Special population group	Community	Partnership	16 months	Control: STD clinics	Non-randomised	Sex workers in treatment (n=110) and	STD/HIV knowledge score	Population	Parameter estimates and standard errors (SE) from random-	Serious

	West Bengal	to impact HIV/STD-related knowledge and skills, in addition to providing treatment and condoms. The programme also diffused rights-based messages to motivate change, building social support and community solidarity, mobilizing political participation to build social capital to enhance advocacy, and diffusing new norms for savings and alternative income enabled by a micro-finance service.				(2000-2001)	established, and provision of peer education	controlled	control (n=110) recruited at baseline			effects linear regression: intercept, 6.7, SE 0.35<br>Know at least one STD 48.5 (14.4, 163)<br>Condoms prevent STDs 23.22 (7.69, 70.3)<br>Condoms prevent AIDS 23.3 (11.2, 48.1)<br>At risk for STDs 6.5 (3.28, 12.9)<br>Important condom decision-maker 24.7 (11.0, 55.6)<br>Can refuse client 9.5 (4.82, 19.0)	
**Water, Sanitation and Hygiene**													
Freeman (2012)	Rural and peri-urban Andhra Pradesh	Representatives from a private company gave presentations to SHG members about sources and risks of contaminated drinking water and methods to effectively treat their water at home, with a demonstration of their company's water filter. After the SHG could take out a loan to buy a filter.	SHG	Group	Informing	18 months (2009-2010)	Female members of an SHG that offered loans for filters but who had not purchased the filter.	Case-control study. A case was defined as a female SHG member whose household had acquired a filter.	SHG women from villages within 1.5 hours radius of central water testing facility. Adopters (n=265) non-adopters(n=247)	Uptake of filter and Thermotolerant Coliform (TTC) count per 100ml	Group	Of the 67230 members who received the program, 9.8% bought the filter.<br><br>The geometric mean TTC count was 13.7 (95%CI: 9.9–18.8) among adopters, and 44.5 (95%CI: 33.7–58.8) among non-adopters (p=0.01).	Serious
Khush (2009)	Rural Tamil Nadu	12 independent community-level programs that employed similar implementation strategies and were initiated at different time points over 3.5 years, e.g. providing	SHG	Group	Informing	3.5 years (2003-2007)	Control group villages with no intervention	Non-randomised controlled	All households in study villages with a child under five years Control (n=456) and Intervention (n=444)	Water and sanitation infrastructure improvements	Group	48% of intervention households had a new private toilet vs 15% of control;<br><br>26% of intervention households had a new water source compared with 18% of control;	Serious

		households with toilets, taps, renovating handpumps and hygiene education campaigns in the community and schools, repair of school water facilities and a micro-credit scheme to take loans from SHGs to construct sanitation infrastructure.										12% of intervention households had a new private tap vs. 8% of control households<br><br>.	
**Vector-borne diseases**													
Nandha (2012)	Rural Tamil Nadu	Health education in schools by a social scientist and teachers. Students educated community members at fortnightly intervals on elimination of breeding sites of mosquitoes SHGs were trained in environmental management methods for mosquito control, who in turn educated their members in monthly meetings and visited households to ensure prevention of mosquito breeding.	SHG	Community	Informing	12 months (2009-2010)	A single village comparable to intervention village in relation to geographic conditions, filarial prevalence, population structure and economic status.	Non-randomisedcontrolled	All household members in the study villages	The proportion of respondents who provided at least one correct answer to each question of the knowledge test	Population	Knowledge: Breeding sites (I:83.9%; C:48.8%, p<0.05); Mosquito-borne diseases (I:75.8;C:48.8,<0.05); preventive measures (I:83.9; C:48.8, p<0.05); transmission by mosquito bite (I:93.5; C:73.2, p<0.05).<br><br>Intervention area: fewer mosquito breeding sites <0.05; better use of personal protection methods (<0.05), better waste water management use (p<0.05) and were more likely to clean surrounding daily (P<0.05). and reduced per man-hour density of mosquitoes	Serious
**Health expenditure**													
Joshi (2016)	Rural Odisha	The Odisha Rural Livelihood Project (TRIPTI) formed SHGs, Gram Panchayat Level Federations (GPLFs) and provided community Investment Funds to improve	SHG	None	Informing	36 months (2011-2013)	Control Gram Panchayats where the program had not	Non-randomisedcontrolled	Int (n=1152) HH Control (n=1189 HH)	HH expenditure on healthcare	Population	Expenses per capita: healthcare (annual)-Treatment- $839.6 vs control- $948, p value=0.126	Moderate

		credit access and promote the productive use of these funds.					been implemente d						
**Mental health**													
Anand (2019)	Rural Uttar Pradesh	Mahila Vikas Pariyojana SHGs with regular meetings focused on the collection of these savings. Regular monthly meetings provide an opportunity to take part in financial and educational activities and build mutual support. SHG members sometimes share maternal and neonatal health-related information by themselves or after training by specialists.	SHG	None	Informing	Not reported	No group	Cross-sectional survey with propensity score matching	5433 members and non-members	15 capability indicators	Group	No changes in capabilities more directly related to health: (1) health limits activities; (2) lost sleep because of worry (mental health).	Moderate

* Outcomes are primary unless specified. †Risk of Bias assessed using ROBINS-1 (Sterne 2016)

Supplementary Table 4: Non-Experimental (Quantitative and Qualitative) Studies

First author (year of publication)	Title	Setting	Objective	Data collection methods	Group type	Key findings
Acharya (2014)	Knowledge on health and nutrition among self-help groups affects the nutritional status	Odisha	To assess the nutritional status of SHG members in tribal areas of Odisha, and whether joining a Self Help Group (SHG) improved nutrition and health knowledge	Cross-sectional quantitative survey	SHG	Low education, scanty income, deficient savings, and meagre assets are barriers to attaining health and improving nutritional status amongst SHG women
Agarwal (2008)	Strengthening functional community provider linkages: Lessons from the Indore urban health programme	Madhya Pradesh	To describe an Urban Health Programme which aimed to increase coverage of services and adoption of key health behaviours related to neonatal survival, diarrhoea control, and other child health priorities by improving the capacities of local stakeholders and slum-based groups in health behaviour promotion	Cross-sectional quantitative survey	Open	An urban health programme integrating demand-supply and ward coordination enhanced utilization of services among slum communities and helped improve immunization coverage and other maternal and child health indicators, in a potentially replicable approach
Alcock (2009)	Community-based health programmes: role perceptions and experiences of female peer facilitators in Mumbai's urban slums	Urban Maharashtra (Mumbai)	To explore the role perceptions and experiences of facilitators of peer-led health interventions as change agents in a community setting	Qualitative - focus group discussions, semi-structured interviews and observation s	Open	Peer-led health programmes need to account for the nature of relationships between peer workers and groups, role perceptions of peer leaders and perceptions and expectations of intervention recipients. Conceptual frameworks to describe the relationship between peer facilitators and groups should be based on empirical (street-level) evidence as well as theory. Programmes need to emphasise rapport-building, communication and negotiation skills for peer educators, and consider how recruitment, training and supervision of peer workers can enhance their credibility in the community.
Aruldas (2017)	Care-seeking behaviours for maternal and newborn illnesses among self-help group households in Uttar Pradesh, India	Rural Uttar Pradesh	To understand the processes of recognition and care-seeking for maternal and newborn illnesses; the sequences of actions for care-seeking when families experience maternal and newborn illnesses; and how health interventions using SHG platforms influence care-seeking for mothers and newborns' illnesses	Qualitative interviews focused on illness narratives	SHG	Deep-rooted cultural beliefs and rituals guided care-seeking behaviour. When the onset of illness was during pregnancy, care was sought from health facilities. As the step of care for maternal illness, SHG households went to government facilities, and non-SHG households used home-based care. Home-based care was the first step of care for newborn illnesses for both SHG and non-SHG households; however, SHG households were prompt in seeking care outside of home, and non-SHG households delayed seeking care until symptoms were perceived to be severe.

Avula (2019)	The Jeevika Multisectoral Convergence Pilot in Bihar - A Process Evaluation Report	Bihar	To understand implementation platforms, training and awareness of roles, implementation processes, exposure of SHG households to key messages, and utilization of an intervention with JEEVikA groups in Bihar	Mixed-methods process evaluation	SHG	At the mid-point of this JEEVikA intervention, key intervention platforms for the behaviour change communication were, to a large extent, in place and functional. The staff's knowledge of the aim of the pilot, and of their specific roles and responsibilities and intersections of roles with one another was good. The Behaviour Change Communication (BCC) content was largely accurate and comprehensive, covering much of the material in the ASHA training manuals and providing many of the same messages. New cadres of Community Nutrition Resource Persons (CNRPs) and the Health Sub Committee (HSC) were being trained began working, which will ease the burden on the Community Mobilisers (CMs) and Cluster Coordinators (CCs). In about 65 percent of the SHG meetings observed as part of the process evaluation, health and nutrition topics were discussed. The topics of discussion were dietary diversity, pregnancy and new-born care, breastfeeding, and complementary feeding, and these correspond to the topics on which the CMs received training most recently.
Barman (2016)	How is perceived community cohesion and membership in community groups associated with children's dietary adequacy in disadvantaged communities? A case of the Indian Sundarbans	West Bengal	To examine the association between community cohesion and child nutrition	Quantitative cross-sectional survey	SHG, CBWG	With each increase in the perceived community cohesion score (scale 0-9), a child was 1.31 times more likely to have minimum acceptable diet (95 % CI 1.14, 1.50). The odds of minimum acceptable diet were also higher among children whose mothers had primary education (2.09, 95 % CI 1.03, 2.94).
Baruah (2007)	Assessment of public–private–NGO partnerships: Water and sanitation services in slums	Gujarat	To explore opportunities and constraints faced by non-governmental organizations (NGOs) collaborating with public- and private-sector organizations on developing and delivering housing, water and sanitation programs for low-income urban families living in slums	Qualitative case study – secondary reports, focus group discussions, observations and interviews	Open	Through their participation in Community-based Organizations (CBOs), women have become much more vocal about their problems and have acquired the skills and confidence to interact with municipal authorities. Instances were also recorded of women from upgraded slums giving information and guidance to women from other slums to join the project. SEWA's stature as a development organization of national and international repute played a large part in enabling The Gujarat Mahila<br>Housing SEWA Trust (MHT) to negotiate a pivotal role for itself during interactions with the other partners. Development of infrastructure and the provision of basic amenities have a positive influence not only on health, education and income, but also the social lives and sense of confidence of slum residents.
Bhaird (2012)	The Complexity of Community Engagement: developing staff-community relationships in participatory child education and women's rights intervention in Kolkata slums	West Bengal	To examine how sociocultural factors influenced relationship building between NGO staff and community members, and how this mediated community participation in a child education and women's rights intervention in Kolkata	Qualitative - interviews and focus group discussions	CBWG	The more participatory and community-led an intervention, the less predictable it becomes. In this context, community-based women's groups became very powerful, often using violence as a problem-solving mechanism, thereby disrupting the social fabric of the community. The flexibility needed to gain community acceptance and manage unanticipated events relies on trusting relationships between both communities and staff, and also between staff and donors.

Blanchard (2013)	Community mobilization, empowerment and HIV prevention among female sex workers in south India	Karnataka and Maharashtra	To test the associations between exposure to community mobilisation, empowerment, and health-related outcomes (condom use, violence and service use) among sex workers in Karnataka and Maharashtra	Quantitative cross-sectional survey	SPG	Engagement with HIV programs and community mobilization activities was associated with different domains of empowerment. Power within (a measure of self-esteem and confidence) and power with (a measure of collective identity and solidarity) were positively associated with more program contact (p <0.01 and p < 0.001 respectively). These measures of empowerment were also associated with outcomes of "personal transformation" in terms of self-efficacy for condom and health service use (p < 0.001). Collective empowerment (power with others) was most strongly associated with "social transformation" variables including higher autonomy and reduced violence and coercion, particularly in districts with programs of longer duration (p <0.05). Condom use with clients was associated with power with others (p < 0.001), while power within was associated with more condom use with regular partners (p <0 .01) and higher service utilization (p < 0.05).
Blankenship (2008)	Power, community mobilization, and condom use practices among FSW in AP, India	Andhra Pradesh	To analyse the association between power and condom use practices among female sex worker (FSW); to analyse extent to which exposure to a local community mobilization intervention affects these associations	Quantitative cross-sectional survey	SPG	Control over both the type of sex [adjusted odds ratio (AOR) 1.70, 95% confidence interval (CI) 1.23–2.34] and the amount charged (AOR 1.56, 95% CI 1.12–2.16), and economic dependence (AOR 0.54, 95% CI 0.35–0.83) are associated with consistent condom use as is programme exposure (AOR 2.09, 95% CI 1.48–2.94). The interaction between programme exposure and collective agency was also significant. Among respondents who reported both programme exposure and high levels of collective agency, the odds ratio of consistent condom use was 2.5 times that of other FSW.
Blankenship (2014)	Challenging the stigmatization of female sex workers through a community-led structural intervention: learning from a case study of a female sex worker intervention in Andhra Pradesh, India	Andhra Pradesh	To contribute to the growing set of case studies analysing the implementation of Community-led structural interventions (CLSIs) to promote HIV prevention among FSW	Qualitative – ethnography with formal and informal interviews	SPG	The CLSI, through its participation in the government-sponsored AIDS education program raised awareness of Community-led structural interventions (CLSIs) among FSW and mobilised them. The CLSI also organized an alternative public rally, outside of but parallel to the government program, where they reframed FSW not as the carriers of HIV but as public health workers combating it. CLSIs for HIV prevention among FSW are implemented in a context of inequality that constrains their actions, but they can still employ strategies that have the potential to transform that context.
Chakravarty (2012)	Health care and women's empowerment: the role of SHGs	Jharkhand	To estimate the level of health care services provided by the SHGs and the awareness and satisfaction level of their members	Qualitative – interviews and focus group discussions	SHG	SHGs can play a role in creating awareness of health issues through group meetings with women, by holding specific capacity-building trainings on health issues and facilitating exposure to important up-to-date medical information. A substantial influence on women's health and empowerment can only be achieved when these activities are taken up with a view to improving the public provision of health care facilities and accessibility.
Chandrashekhar (2019)	Cost and cost-effectiveness of health behaviour change interventions implemented with self-help groups in Bihar, India	Bihar	To assess the cost effectiveness in terms of cost per neonatal death averted and life year saved resulting from phase 1 of the Parivartan program	Decision modelling and cost-effectiveness analysis	SHG	The cost of forming an SHG group in Bihar was US$254 and that of reaching a woman within the group was $US 19. The unit cost for delivering health interventions through the Parivartan program was US$148 per group and US$11 per woman reached. During an 18 month period, Parivartan program reached around 17,120 SHGs and an estimated 20,544 pregnant women resulting in an estimated prevention of 23 neonatal deaths at a cost of US $3,825 per life year saved.

Dongre (2007)	A comparison of HIV/AIDS awareness between self-help group leaders and other women in the villages of Primary Health Centre, Anji.	Maharashtra	To examine levels of HIV knowledge among SHG leaders vs other women in villages served by one primary health centre.	Quantitative cross-sectional survey	SHG	The leaders of SHGs had better levels of education and awareness about HIV/AIDS than other women in the village. Considering the significant high level of awareness regarding HIV/AIDS, the leaders of women's self-help groups could act as potential resource persons for the delivery of health education to other women.
Dongre (2009)	A Community Based Approach to Improve Health Care Seeking for Newborn Danger Signs in Rural Wardha, India	Maharashtra	To examine the effect of health education and community mobilization intervention on health care seeking of families with sick newborns and explore reasons for changes amongst mothers	Cross-sectional	SPG	There was a significant improvement in mothers' knowledge regarding newborn danger signs. About half of mothers got information from Community-led interventions for Child Survival (CLICS) *doot* (female community health worker). The monitoring over three years period showed encouraging trend in level of awareness among pregnant women. After three years, the proportion of mothers giving no treatment/home remedy for newborn danger signs declined significantly. However, there was significant increase in mothers' healthcare-seeking from private health care providers for sick newborns.
Euser (2012)	Pragati: an empowerment programme for female sex workers in Bangalore, India	Karnataka	To describe the effects of a broad empowerment programme among female sex workers (FSWs) in Bangalore, India, which seeks to develop the capacities of these women to address the issues that threaten their lives and livelihoods	Quantitative process evaluation with implementation, coverage and cost data	SPG	Between 2005 and 2010, the number of women who received help from a crisis response team increased, more women participated in alcohol de-addiction programmes, and the number of saving accounts and distributed microfinance loans was expanded. Furthermore, condom use increased over time, and more FSWs were treated for STIs. In contrast, the number of Sexually Transmitted Infections (STIs) and the STI incidence rate increased over time.
Feldman (2015)	Women's Political Participation and Health: A Health Capability Study in Rural India	Uttar Pradesh	To use a health capability framework with four domains (agency—participation, autonomy, self-efficacy, and health systems) to understand dimensions of health agency and illuminate how local political economies affect health.	Qualitative – semi structured interviews, focus group discussions	Open	Better understanding of cultural norms surrounding autonomy, the local infrastructure and health systems and male and female perceptions of political participation and self-efficacy are needed to improve women's health agency. For a community based participatory health intervention to improve health capabilities effectively, explicit strategies focussed on health agency should be as central as health indicators.
Feruglio (2018)	The challenges of institutionalizing community-level social accountability mechanisms for health and nutrition: a qualitative study in Odisha, India	Odisha	To examine how community accountability mechanisms have sought to strengthen community-level nutrition and health services (Integrated Child Development Services and National Rural Health Mission) when institutionalised at scale	Qualitative – in-depth interviews and focus group discussions	CBWG + SHG	The degree of effectiveness of different groups in strengthening accountability varied depending on their ability to offer meaningful avenues for participation of their members and empower women for autonomous action. In most of the mechanisms, community participation is very weak, with committees largely controlled by the frontline workers who are supposed to be held to account. However, SHGs showed real levels of autonomy and collective power. Despite not having an explicit accountability role, these groups were nevertheless effective in advocating for better service delivery and the broader needs of their members to a level not seen in institutional committees.
Gailits (2019)	Women's freedom of movement and participation in psychosocial support groups: qualitative study in northern India	Uttarakhand	To examine the factors influencing women's participation in psychosocial support groups, within an approach where community members work together to collectively strengthen their community's mental health	Qualitative – key informant interviews and focus group discussions	SPG	Mental health access and gender inequality are inseparable in the context of Northern India, and women's mental health cannot be addressed without first addressing underlying gender relations that prevent participation in support groups. Community-based mental health programs are an effective tool and can be used to strengthen communities collectively; however, attention towards the gender constraints that restrict women's freedom of movement and their ability to access care is required.

George (2018)	Can community action improve equity for maternal health and how does it do so? Research findings from Gujarat, India	Gujarat	To examine the equity effects of community action for maternal health led by Non-Government Organizations (NGOs) on facility deliveries	Mixed methods – qualitative data using project documents and interviews and quantitative data on self-reported use of services	Open, CBWG	The study found substantial increases in receipt of information of entitlements and utilization of antenatal and delivery care, and a switch from private facilities to public ones among the most vulnerable. Implementation required: a) alignment among NGO organizational missions; b) participatory development of project tools; c) repeated capacity building and; d) government interest in improving utilization and recognition of NGO contributions.
Gopalan (2007)	Microfinance and its contributions to health care access: a study of self-help groups (SHGs) in Kerala	Kerala	To understand the role played by self-help groups in Kerala vis-à-vis health	Qualitative – individual interviews	SHG	In order to obviate the difficulties (like inability to repay the loans regularly) experienced by the extremely poor members of microfinance institutions, it is necessary to make the terms and conditions of savings and borrowings more poor-friendly than they are at present. Setting up of a welfare fund at the SHG level to address emergency medical needs is essential. SHG members are often willing to participate if their contributions are supplemented by a government subsidy. Inter-sectoral coordination, by keeping microfinance mechanism as the pivot or by incorporating microfinance mechanism can ensure an easy, appropriate, affordable and effective service delivery at the doorstep.
Gopalan (2015)	Leveraging Community-Based Financing for Women's Nonmaternal Health Care: Experiences of Rural Indian Women	Odisha	To explore the potential of community-based financing for nonmaternal health care through a demand-side qualitative assessment among rural Indian women	Qualitative – focus group discussions	CBWG	Community-based financing provided financial access and risk protection for women's non-maternal health care, though not adequately. Schemes covering outpatient care (or mild illnesses) provided relatively more financial access. The major determinants of their restricted financial access were limited sum assured, noncomprehensive coverage of services, exclusion of elderly women, and the low priority thay households gave to non-maternal health care. Community-based financing requires relevant structural changes along with demand-side behavioural modifications to ensure optimal attention to women's nonmaternal health care.
Gupta (2009)	Impact of a Health Education Intervention Program Regarding Breast Self-Examination by Women in a Semi-Urban Area of Madhya Pradesh, India	Madhya Pradesh	To determine the awareness and practice regarding Breast Self- Examination (BSE) in women; to assess the impact of health education on awareness and practice of BSE; to identify other factors affecting on the awareness and practices of BSE	Quantitative pre-post uncontrolled intervention study	SPG	The study found significant improvement in knowledge regarding all aspects of BSE of the intervention group. After the intervention program, 590 (59%) women had good knowledge and among them 90.7% practiced BSE compared to 0% at pre-test. An overall increase in the awareness of BSE practice (43% to 53%) was observed in the study group after intervention.

Gupta (2015)	Empowerment and engagement of SHGs against RTI/STI in Karnataka, India: an interventional study	Karnataka	To evaluate the effect of multi-centric action research to sensitize, mobilize and engage women through SHGs, to improve reproductive health, particularly Reproductive Tract Infection (RTI)/STIs and cervical cancer	Quantitative pre-post intervention study, (no baseline variables) and qualitative focus group discussions	SHG	The Intervention was effective in improving women's awareness about RTI/STIs, correct knowledge about white discharge, capability to identify the symptoms of RTI/STI and health seeking behaviour of the respondents. There was no observed change in prevalence of RTI/STIs.
Hamal (2018)	How does social accountability contribute to better maternal health outcomes? A qualitative study on perceived changes with government and civil society actors in Gujarat, India	Gujarat	To explore social accountability mechanisms in relation to maternal health, the factors they address and how the results of these mechanisms are perceived	Qualitative – in-depth interviews and focus group discussions	CBWG	Social accountability mechanisms influenced structural determinants (governance, policy, health beliefs, women's status) and intermediary determinants (social capital, maternal healthcare behaviour and availability, accessibility) and quality of health service delivery system. These further positively influenced the increased use of maternal health services. The social accountability mechanisms, through the process of information, dialogue and negotiation, particularly empowered women to make collective demands of the health system and brought about changed perceptions of women among actors in the system. It improved relations between women and the health system in terms of trust and collaboration, and generated appropriate responses from the health system to meeting women's groups' demands.
Hunt (2001)	Pathways to empowerment? Reflections on microfinance and transformation in gender relations in South Asia	Bihar (and Bangladesh)	To critically reflect on the pathways between microfinance and empowerment, including mobility and violence against women	Qualitative – interviews	SHG	Microfinance must be re-assessed in the light of evidence that the poorest families and the poorest women are not able to access credit. A range of microfinance packages is required to meet the needs of both the poor and the poorest. Development agencies need to acknowledge that microfinance does not directly or automatically lead to women's empowerment and gender transformation. More reflection and documentation is needed on pathways to empowerment, and on programme strategies that assist women to take greater control of decision-making and life choices.
Jejeebhoy (2018)	Preventing violence against women and girls in Bihar: challenges for implementation and evaluation	Bihar	To describe mechanisms and challenges through which the *Do Kadam* programme brought about change in outcomes that it sought to affect	Mixed methods process evaluation	SHG	Contextual challenges to the intervention success included lack of leadership skills of those delivering the intervention and the gap between expected responsibilities and activities of government platforms and reality. Implementation challenges were encountered in reaching men and boys, younger women and the community at large and ensuring their regular attendance; and in maintaining the fidelity of the intervention activities. Evidence-supported dialogue on these challenges and how best to anticipate and address them is essential.
Kadiyala (2016)	Adapting Agriculture Platforms for Nutrition: A Case Study of a Participatory, Video-Based Agricultural Extension Platform in India	Odisha	To examine the process of integrating Maternal, Infant and Young Child Nutrition (MIYCN) into the existing low-cost, participatory, video-based agricultural extension platform targeted to women's self-help groups and compare the development and delivery of agriculture and nutrition content; To assess the	Qualitative – in-depth interviews, structured observations, knowledge tests and questionnaires	SHG	Nutrition intervention were well-received by rural communities and viewed as complementary to existing frontline health services. However, compared to agriculture, nutrition content required more time, creativity, and technical support to develop and deliver. Experimentation with promoted nutrition behaviours was high but sharing of information from the videos with non-viewers was limited. There is a need for collaboration with existing health services; continued technical support for implementing partners; engagement with local cultural norms and beliefs; empowerment of women's group members to champion nutrition; and enhancement of message diffusion mechanisms to reach pregnant women and mothers of young children at scale.

			viability of promoting nutrition-specific actions through the platform, including acceptance and trial of promoted behaviours and diffusion of key messages; and to assess synergies with government health and nutrition services			
Kaur (2017)	Evaluation of a women group led health communication program in Haryana, India	Haryana	To describe the functionality and reach of Sakshar Mahila Smooh (SMS) as well as Auxiliary Nurse Midwives (ANM) and rural women's perceptions of the SMS	Cross-sectional	Open	Out of 2009 villages, 1732 (86%) had functional SMSs. In three years, Most ANMs opined that SMSs are better health communicators. SMS members were aware about their roles and responsibilities. The majority of village women reported that SMS carry out useful health education activities. The characteristics of SMS members were similar, but program performance was better in districts where health managers were proactive in program planning and monitoring.
Kermode (2008)	Some peace of mind: assessing a pilot intervention to promote mental health among widows of injecting drug users in north-east India	Manipur and Nagaland	To learn about women's perspectives on mental health and well-being and the links between mental health and HIV; to assess changes in the women's quality of life and mental health during the course of the intervention; to assess changes in engagement in HIV risk behaviours; to describe the process and outcome of the intervention from the perspective of the women.	Mixed methods process evaluation – questionnaire survey and focus group discussions	SPG	Widows of injecting drug-users, organized into participatory action groups showed significant improvements in quality of life, mental health and experience of somatic symptoms, and the women told stories reflecting a range of significant changes. A participatory approach to mental health promotion can have a positive impact on the lives of vulnerable women and has the potential to contribute to HIV prevention.
Kethineni (2016)	Combating Violence against Women in India: Nari Adalats and Gender-Based Justice, Women & Criminal Justice	Karnataka	To examine the effectiveness of Nari Adalats as an alternative avenue for women seeking justice; To identify the role of Mahila Samakhyas in empowering rural and disadvantaged women in India	Qualitative – interviews	CBWG	Nari Adalats (women's courts) exercise broad authority to investigate and address a wide range of domestic violence cases in India. The Mahila Samakhya (women's federation) serves as an advocacy group and provides shelter, legal assistance, and social help as well as education for victims of domestic violence. The Mahila Samakhya is committed to empowering women who cannot find justice through formal governmental means.
Krishnan (2012)	An Intergenerational Women's Empowerment Intervention to Mitigate Domestic Violence: Results of a Pilot Study in Bengaluru, India	Karnataka	To present findings on intervention feasibility, acceptability and safety from a pilot study of 20 Daughter-in-Law - Mother-in-Law (DIL-MIL) dyads in urban low income communities in Bengaluru	Qualitative – focus group discussions and in-depth interviews	SPG	A family-based approach to violence prevention is highly promising. With increased awareness and knowledge of gender inequities, violence, and health, enhanced relationship skills, and peer support, intergenerational relationships can be safely mobilized to mitigate domestic violence.

Kumar (2007)	Health inequity and women's self-help groups in India: The role of caste and class	Bihar	To review the scope and limitations of SHGs in improving women's health using the example of Bihar, India, and in particular to assess the extent to which SHGs can be involved in attaining better health for women and children by exploring the role of caste and class in access to health services	Qualitative – field surveys, interviews, focus group discussions and case studies	SHG	Caste imposes serious limitations on the extent to which SHGs can be used in improving women's health. Women's health is very much dependent on existing gender relations, and their interaction with income, education and general standards of living. SHG programmes are functioning in a vacuum without addressing these contextual issues, severely constrained in being able to have a significant effect on women's health. Decentralisation and local accessibility of public health facilities is a pressing requirement to advance the health of poor and marginalised women.
Kumar (2009)	Participation in Self-help Group Activities and Its Impacts: Evidence from South India	Tamil Nadu	To compare household income and expenditure among households with women SHG members and households with women who do not belong to SHGs	Quantitative cross-sectional survey	SHG	SHGs generate substantial income and have significance in the household. The quantity and quality of food consumed, the health of household members and children's education improve. Institution building contributes greatly towards improving household welfare.
Kumar (2015)	Enculturating science: Community-centric design of behaviour change interactions for accelerating health impact	Uttar Pradesh	To describe a systems approach for community-centric design of interactions, highlighting key principles for achieving intrinsically motivated, sustained change in social norms and family health behaviours, elucidated with progressive theories from a range of disciplines.	Qualitative – case study	Open	Behaviour change can be achieved when biomedical and traditional socio-cognitive systems are understood to co-develop solutions to address a health issue. This requires recognition of the fact that one is not dealing with individuals, but community systems that were designed over generations keeping in mind a certain worldview and a common social purpose. In order to design scientifically guided effective interactions, it is important to understand the causal mechanisms and underlying system that govern these behaviours.
Kumar (2019)	Social networks, mobility, and political participation: The potential for women's self-help groups to improve access and use of public entitlement schemes in India	Madhya Pradesh, Odisha, Chhattisgarh, Jharkhand and West Bengal	To examine the potential for women's SHGs to improve access to and use of public entitlement schemes	Quantitative cross-sectional survey	SHG	SHG members are more politically engaged and more likely to know of certain public entitlements than non-members. They are significantly more likely to access a greater number of public entitlement schemes. SHG members have wider social networks and greater mobility as compared to non-members. SHGs can enforce accountability amongst public entities and demand what is rightfully theirs. SHGs themselves cannot be expected to increase knowledge in absence of an external agency.
Long (2013)	Determinants of better health: a cross-sectional assessment of positive deviants among women in West Bengal	West Bengal	To identify factors associated with positive health outcomes among women with primary education or less (positive deviants)	Quantitative cross-sectional survey	SHG	Positive deviants in this context are shown to be women who are able to earn an income, who have access to information through media sources, and who, despite little schooling, have marginally higher levels of formal education that lead to improved health outcomes.

Mohindra (2008)	Can microcredit help improve the health of poor women? Some findings from a cross-sectional study in Kerala, India	Kerala	To examine associations between female participation in SHGs, and women's health in Kerala	Quantitative cross-sectional survey	SHG	Compared to non-participants living in a household without a SHG member, the odds of facing exclusion is significantly lower among early joiners, women who were members for more than 2 years (OR = 0.58, CI = 0.41–0.80), late joiners, members for 2 satisfaction compared to non-members (OR = 0.52, CI = 0.30–0.93; OR = 0.32, CI = 0.14–0.71). No associations were found between SHG participation and self-assessed health or exposure to health risks. The relationship between SHG participation and decision-making agency is unclear.
Morrison (2019)	Exploring the equity impact of a maternal and newborn health intervention: a qualitative study of participatory women's groups in rural South Asia and Africa	Jharkhand and Odisha	To understand the mechanisms that led to the equitable impact of the Participatory Learning and Action (PLA) approach across socioeconomic strata in 4 sites in India, Nepal, Bangladesh and Malawi	Qualitative – focus group discussion, interviews, key informant interviews	Open	Participatory learning and action led to increased knowledge, confidence to act, and acceptability of recommended practices. The equitable behavioural effects were facilitated by the accessibility, relevance, and engaging format of the intervention across socioeconomic groups, and by reaching-out to parts of the population usually not accessed. A participatory approach improved health behaviours across socioeconomic strata in rural communities, around issues for which there was a knowledge deficit and where simple changes could be made at home.
Panda (2015)	Mobilizing community-based health insurance to enhance awareness & prevention of airborne, vector-borne & waterborne diseases in rural India	UP and Bihar	To evaluate the effect of a health education program -- campaigns with SHGs -- on airborne, vector borne and waterborne disease	Quantitative uncontrolled pre-post intervention study	SHG	The study found significant increases both in awareness (34%, p<0.001) and in preventive practices (48%, P=0.001), suggesting that the awareness campaign was effective. However, average practice scores (0.31) were substantially lower than average awareness scores (0.47), even in post campaign. Awareness and preventive practices less prevalent for vector-borne diseases than in airborne and waterborne diseases. Education was positively associated with both awareness and practice scores. The awareness scores were positive and significant determinants of the practice scores, both in the pre- and in the post-campaign results. Affiliation to Community-based health intervention (CBHI) had significant positive influence on awareness and on practice scores in the post-campaign period.
Prabhakaran (2016)	Impact of Community-led Total Sanitation on Women's Health in Urban Slums: A Case Study from Kalyani Municipality	West Bengal	To understand the impact of improved sanitation and specifically of the Community-led total sanitation (CLTS) process on women's physical health in terms of reduction in disease burden; and the social and psychological wellbeing of women in selected slums of Kalyani; to understand the impact of the CLTS process on aspects of women's empowerment and its effect on women's wellbeing and overall health in selected Open Defaecation Free (ODF) slums of Kalyani; to understand the external environmental factors that have played a key role in improving sanitation in	Qualitative – focus group discussions, personal interviews, key informant interviews	Open	Political will. commitment from local institutional actors, the ability to mobilise resources and capacity to work with the community are all needed to achieve long-term change with CLTS. Institutional actors such as politicians, administrators, health workers, engineers and contractors can play in achieving successful outcomes, not as direct implementers of the programme or as providers of infrastructure, but as facilitators supporting the community to design and implement its own initiatives. The community has to take ownership and accept accountability for their sanitation and hygiene behaviour and practices. Collective community demand and action in activating and strengthening formal health delivery systems and integrating health programmes into sanitation initiatives.

			Kalyani and therefore the health of women			
Raghavendra (2014)	Nature of activities organised by self-help groups formed by two non-governmental organisations for the integrated development of the members and the community	Karnataka	To describe the nature of activities organised by SHGs formed by 2 NGOs in the state	Qualitative – focus group discussions, observations	SHG	The SHGs carried out 31 different types of activities, indicating that the women could do a wide range of activities if they were organised and trained. SHGs are an appropriate forum for rural women to expose themselves to mainstream economic sphere and become economically independent as well as participate in decision making process in their respective families.
Rajendran (2010)	Role of community empowerment in the elimination of lymphatic filariasis in south India	Tamil Nadu	To examine how a community empowerment-focussed IEC intervention worked to improve use of mass drug administration to eliminate lymphatic filariasis	Cohort and focus group discussions	SHG	After four rounds of mass drug administration (MDA), there was a significant decline in the filarial infection variables. The microfilaraemia and antigenaemia declined by 59% and 67% respectively. The transmission indices lowered by 89% and 94% (in resting and landing catch of mosquitoes respectively). The decline in these variables, with a drug consumption rate of >80% was achieved due to the effective Information Education Communication (IEC) campaigns prior to each MDA. After 4 MDAs almost 97% of the respondents were aware of lymphatic filariasis. SHGs and school students were observed to be integral to MDA campaigns for the enhancement of drug compliance, thus leading to lymphatic filariasis elimination.
Rao (2011)	Community-Based Mental Health Intervention for Underprivileged Women in Rural India: An Experiential Report	Karnataka	To share experiences from a project that integrates a mental health intervention within a developmental framework of microcredit activity for economically underprivileged women in rural India	Qualitative – focus group discussions	SHG	Women in the mental health intervention group reported reduction in psychological distress and bodily aches and pains. The majority reported that the quality of their sleep had improved with regular practice of relaxation and that sharing their problems in the group had helped them to unburden. The social support extended by the members to each other, made them feel that they were not alone and could face any life situation. Adding the mental health intervention to the ongoing economic activity made a positive difference in the lives of the women. Addressing mental health concerns within livelihood initiatives can help to enhance both economic and social capital in rural poor women.
Rath (2010)	Explaining the impact of a women's group led community mobilisation intervention on maternal and newborn health outcomes: the Ekjut trial process evaluation	Jharkhand and Odisha	To report process evaluation data from the Ekjut trial of a Participatory Learning and Action (PLA) cycle with women's groups	Mixed-methods process evaluation	Open	Participatory interventions with community groups can influence maternal and child health outcomes if key intervention characteristics are preserved and tailored to local contexts. Scaling-up such interventions requires a detailed understanding of the way in which context affects the acceptability and delivery of the intervention; planned but flexible replication of key content and implementation features; strong support for participatory methods from implementing agencies.
Reshmi (2019)	Context for layering women's nutrition interventions on a large scale poverty alleviation program: Evidence from three eastern Indian states	Bihar, Chhattisgarh, Odisha	To describe the scenario or context prior to layering of women's nutrition interventions on NRLM platforms	Quantitative cross-sectional survey	SHG	BMI indicated at least 45% mothers were undernourished irrespective of their enrolment in SHGs. Higher proportion of SHG members (77%-87%) belonged to food insecure households than non-members (66%-83%). Current use of family planning (FP) methods was excruciatingly low (8.2%-32.4%) in all states but positively skewed towards SHG members.

Ruducha (2019)	Measuring coordination between women's self-help groups and local health systems in rural India: a social network analysis	Uttar Pradesh	To assess how health services coordination and emergency referral networks between SHGs and local health systems, along with other key stakeholders, changed over the course of a 2-year learning phase of the project using social network analysis (SNA)	Quantitative uncontrolled pre-post intervention study with cross-sectional surveys	SHG	The health services coordination and emergency referral networks increased in density and the number of connections between respondents as measured by average degree centrality have increased, along with more diversity of interaction between groups. The network expanded relationships at the village and block levels, reflecting the rise of bridging social capital. The accredited social health activist, a village health worker, occupied the central position in the network, and her role expanded to sharing information and coordinating services with the SHG members.
Saggurti (2013)	Community collectivization and its association with consistent condom use and STI treatment-seeking behaviours among female sex workers and high-risk men who have sex with men/ transgenders in Andhra Pradesh, India	Andhra Pradesh	To examine community collectivisation among FSWs (female sex workers) and HR-MSM (high risk Men who have sex with men), and measure its association with select outcome indicators	Quantitative cross-sectional survey	SPG	High levels of collective efficacy (adjusted OR: 1.3, 95% CI: 1.11.7) and collective action (adjusted OR:1.3, 95% CI: 1.11.8) were associated with consistent condom use (CCU) with regular clients among FSWs. Among HR-MSM, participation in a public event (adjusted OR: 2.7, 95% CI: 2.03.6) and collective efficacy (adjusted OR: 1.9, 95% CI: 1.52.3) were correlated with condom use with paying partners.
Saha (2013)	The effect of Self-Help Groups on access to maternal health services: evidence from rural India	All India	To assess the impact of the presence of SHGs on maternal health service uptake	Secondary analysis of a quantitative cross-sectional survey (District-Level Household Survey)	SHG	Respondents from villages with a SHG had a 19% increased odds (OR: 1.19, CI: 1.13-1.24) of delivering in a health facility, increased knowledge of(OR: 1.48, CI 1.39 – 1.57) and utilization of family planning products and services (OR: 1.19, CI 1.11 – 1.27). These results were significant after controlling for individual and village-level heterogeneities.
Sanyal (2015)	Recasting Culture to Undo Gender: A Sociological Analysis of Jeevika in Rural Bihar, India	Bihar	To understand how Jeevika induced large scale cultural change in Bihar	Qualitative –interviews, focus group discussions, non-participant observation, structured interviews	SHG	Jeevika created new "cultural configurations" by giving economically and socially disadvantaged women access to a well-defined network of people and new systems of knowledge, which changed women's habitus and broke down normative restrictions constitutive of the symbolic boundary of gender.
Sethi (2017)	Partnering with women collectives for delivering essential women's nutrition interventions in tribal areas of eastern India: a scoping study	Odisha, Jharkhand and Chhattisgarh	To examine the feasibility of engaging women collectives in delivering a package of women's nutrition messages/services as a funded stakeholder in three tribal-dominated districts	Mixed methods – secondary of quantitative data, interviews and focus group discussions	SHG	Limited targeting of pre-pregnancy period, delays in first trimester registration of pregnant women, and low micronutrient supplementation supply and awareness issues impact women's nutrition. SHGs with organisational readiness for receiving and managing grants for income generation and community development activities varied from 41% to 94%.

Sinha (2006)	Self-help groups in India: a study of the light and shades	Andhra Pradesh, Karnataka, Odisha, Rajasthan	To examine the efficiency of SHGs in their financial transactions; their sustainability; the extent to which they are able to take social action; who benefits from these actions; who drops out and why	Qualitative – semi-structured interviews, transect walks, informal interviews, focus group discussions	SHG	Understanding the effectiveness of SHGs – whether in terms of financial or social empowerment - requires greater clarity of vision and objectives and a systematic approach to building capacity and providing guidance. There is a need to define objectives for creating SHGs based on the needs of its members, as well as understanding the impact of social networks and local politics. Additionally, there is a need to deliberate on the extent and length of support provided to SHGs as well as dealing with members dropping out or SHGs becoming defunct.
Sinha (2017)	Economic evaluation of participatory learning and action with women's groups facilitated by Accredited Social Health Activists to improve birth outcomes in rural eastern India	Jharkhand and Odisha	To assess the cost-effectiveness of a PLA intervention facilitated by ASHAs to improve neonatal outcomes	Used cluster RCT data (Tripathy 2016) and cost data collected	Open	The incremental cost of the intervention was USD 83 per averted disability-adjusted life years (DALY) (USD 99 inclusive of VHSNC strengthening costs), and the incremental cost per newborn death averted was USD 2545 (USD 3046 inclusive of Village Health, Sanitation and Nutrition Committee (VHSNC) strengthening costs). The intervention was highly cost-effective according to WHO threshold, as the cost per life year saved or DALY averted was less than India's Gross Domestic Product (GDP) per capita. The robustness of the findings to assumptions was tested using a series of one-way sensitivity analyses. The sensitivity analysis does not change the conclusion that the intervention is highly cost-effective.
Swamy (2013)	Women Financing and Household Economics	Karnataka	To assess whether women's financing through groups improves food security (through measuring food expenditure) and standard of living, especially of women and vulnerable caste groups	Quantitative uncontrolled pre-post intervention study with cross-sectional surveys	SHG	Access to finance through groups has significant impacts on poor families' food security and non-food expenses. The study has evidenced significant outreach of impact of women financing in terms of physical as well as qualitative factors on the socially weaker sections of the society such as Women, Scheduled Castes /Scheduled Tribes and Other Backward Classes category of the poor.
Van Rompay (2008)	Empowering the people: Development of an HIV peer education model for low literacy rural communities in India	Tamil Nadu	To describe a HIV peer education model to educate and empower low-literacy communities in a rural district	Mixed-methods process evaluation with pre- and post-test surveys and focus group discussions	SHG	Using established networks (such as community-based organizations already working on empowerment of women) and training women's SHG leaders and barbers as peer educators was an effective and culturally appropriate way to disseminate comprehensive information on HIV/AIDS to low-literacy communities. Similar models for reaching and empowering vulnerable populations should be expanded to other rural areas.

Supplementary Figure 1: Location of included studies, by state

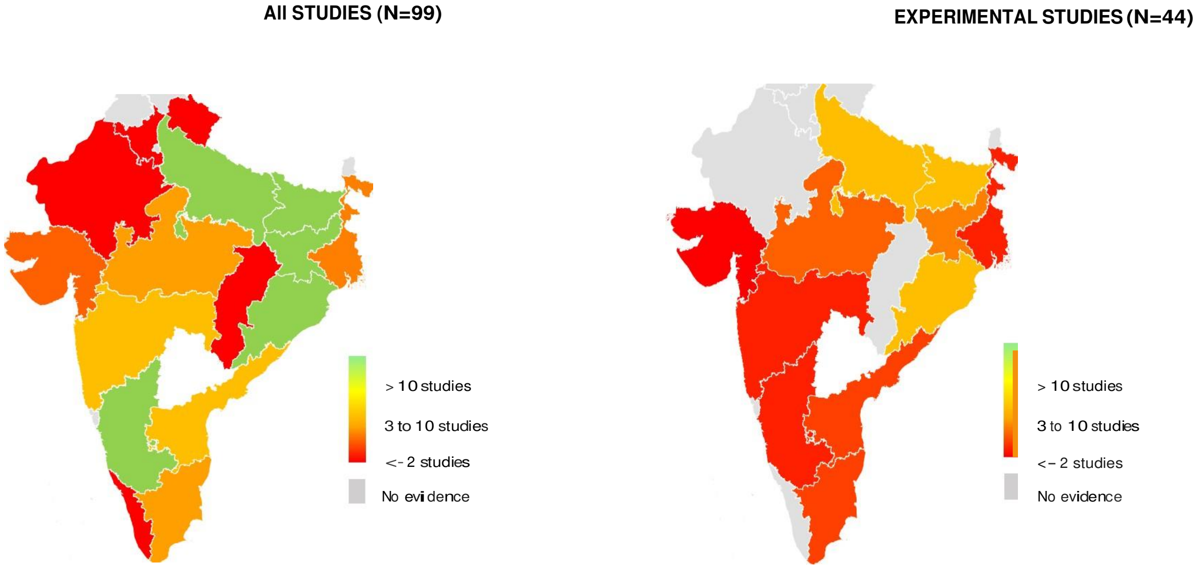

Supplement: Supplementary data [file bmjgh-2020-003304supp001.pdf]
